# Elevated pre-activation basal level of nuclear NF-κB in native macrophages accelerates LPS-induced translocation of cytosolic NF-κB into the cell nucleus

**DOI:** 10.1038/s41598-018-36052-5

**Published:** 2019-03-14

**Authors:** Alexander V. Bagaev, Anastasiya Y. Garaeva, Ekaterina S. Lebedeva, Alexey V. Pichugin, Ravshan I. Ataullakhanov, Fazly I. Ataullakhanov

**Affiliations:** 1 National Research Center – Institute of Immunology Federal Medical-Biological Agency of Russia, Moscow, Russia; 2 Center for Theoretical Problems of Physicochemical Pharmacology, Russian Academy of Sciences, Moscow, Russia; 3 National Scientific and Practical Center of Pediatric Hematology, Oncology and Immunology, Moscow, Russia; 4 Department of Physics, Moscow State University, Moscow, Russia; 5 Department of Biology, Moscow State University, Moscow, Russia; 6 Moscow Institute of Science and Technology, Dolgoprudny, Russia

## Abstract

Signaling via Toll-like receptor 4 (TLR4) in macrophages constitutes an essential part of the innate immune response to bacterial infections. Detailed and quantified descriptions of TLR4 signal transduction would help to understand and exploit the first-line response of innate immune defense. To date, most mathematical modelling studies were performed on transformed cell lines. However, properties of primary macrophages differ significantly. We therefore studied TLR4-dependent activation of NF-κB transcription factor in bone marrow-derived and peritoneal primary macrophages. We demonstrate that the kinetics of NF-κB phosphorylation and nuclear translocation induced by a wide range of bacterial lipopolysaccharide (LPS) concentrations in primary macrophages is much faster than previously reported for macrophage cell lines. We used a comprehensive combination of experiments and mathematical modeling to understand the mechanisms of this rapid response. We found that elevated basal NF-κB in the nuclei of primary macrophages is a mechanism increasing native macrophage sensitivity and response speed to the infection. Such pre-activated state of macrophages accelerates the NF-κB translocation kinetics in response to low agonist concentrations. These findings enabled us to refine and construct a new model combining both NF-κB phosphorylation and translocation processes and predict the existence of a negative feedback loop inactivating phosphorylated NF-κB.

## Introduction

Bacterial lipopolysaccharide (LPS) is a classical agonist of TLR4^1^. The innate immune response to bacterial infection is initiated and guided by macrophages, which are key components of the immune system^2^. Macrophages largely determine the effectiveness of first-line defense against infections, producing active radicals, peroxides, cationic peptides, interferons, lysozymes and hydrolytic enzymes^3,4^, while concurrently harboring powerful destructive potential against the host’s own cells. Consequently, such an important protective weapon (unsafe for the host’s own tissues) must be accurately and effectively regulated. The regulation setting should both contribute to the earliest possible detection of microbial substances and be non-responsive to extrinsic noise, having an activation threshold that non-linearly depends on the concentration of agonistic ligands.

Almost all knowledge of TLR4 signaling pathways comes from studies of transformed cell lines^5–9^, with little from primary cells or macrophages. There are many experimental and theoretical studies concerning analysis of exact signaling events occurring upon activation with TLR4 agonists^1,10,11^. LPS binding to TLR4 leads to the activation and translocation of nuclear factor kappa B (NF-κB) transcription factor into the nucleus, which triggers the transcription of target genes^2,12^. Immortalized cell lines are convenient for the study of cell signaling because they can be genetically modified to produce, for example, NF-κB subunits fused with fluorescent proteins, enabling observation of a single cell’s NF-κB dynamics^8,13^. While the general signaling events in cells are consistent, their kinetics, timing and regulation vary across different cell types. For example, mouse fibroblast 3T3 cells^14^ have diminished NF-κB oscillations in comparison to the mouse macrophage-like RAW 264.7 cell line, human epithelial HeLa^15^ or mouse embryonic fibroblast (MEF) cells^5^. These cell types are also affected differently by paracrine cytokines induced after NF-κB activation^8,16,17^. This raises the of question whether primary (non-transformed) macrophages have the same activation features and comply with the NF-κB signaling regulation observed in cancerously transformed and genetically modified cells.

However, getting an answer to this question is not an easy task, because soon after TLR4 receptor dimerization, a multitude of molecular interactions take place that induce NF-κB activation^18^. It is very difficult to understand such a complicated web of reactions without mathematical modeling. Much is known about the mathematical dependencies of protein interactions in the NF-κB signaling pathway. Many models have been designed regarding different cell lines. However, we failed to precisely describe our experimental data using non-transformed primary macrophages with existing mathematical models.

The goal of this study was to systematically and consistently analyze TLR4 activation at a wide range of LPS concentrations in order to mathematically describe the NF-κB kinetic response in primary bone marrow-derived macrophages (BMMϕ). We created an accurate mathematical description of both NF-κB translocation and phosphorylation processes dependent on the concentration of the initiating TLR4 ligand. It is much easier to experimentally measure NF-κB phosphorylation as compared to NF-κB translocation to the cell nucleus. To our knowledge, this is the first attempt to link models of NF-κB nuclear translocation and phosphorylation.

We found that agonist-induced activation of TLR4 signaling and NF-κB translocation in primary macrophages is significantly faster than in immortalized cell lines. Post-LPS activation of NF-κB phosphorylation peaked at 5 min, whereas IκBα degradation and NF-κB nuclear translocation kinetics peaked at 10 min. Signaling kinetics were substantially faster in comparison to the transformed RAW 264.7 macrophage cell line^8^, where NF-κB nuclear translocation peaked at 30 min after LPS stimulation. The most significant differences in signaling kinetics were observed with low concentrations of LPS (~2 ng/ml) inducing slow and weak responses in both primary and transformed macrophages, although responses were faster in the primary macrophages than in RAW cells (50 min vs. 75 min).

Mathematical modeling predicted that constitutive basal NF-κB pre-activation could speed up the initial rate of NF-κB translocation, explaining the differences in experimental kinetics between cells at low LPS concentrations. The existence of a predicted increased level of basal nuclear NF-κB was proven in our experiments. Thus, prior to LPS stimulation, native macrophage baseline nuclear NF-κB levels were as high as 25–35% of the total cellular amount. In contrast, baseline nuclear NF-κB levels in the transformed RAW 264.7 macrophage cell line comprised not more than 5–10% of the total cellular NF-κB. Therefore, we propose a previously unconsidered mechanism for nuclear NF-κB regulation, which can significantly increase the speed of macrophage response, specifically at low concentrations of LPS. This mechanism may be crucially important for pathogen detection near the threshold concentrations.

NF-κB phosphorylation controls NF-κB directed transactivation. Importantly, NF-κB phosphorylation controls transcription in a gene-specific manner^19^. Also, it could be measured more easily in the experimental settings comparing to the nuclear translocation. To extent the NF-κB model to describe pro-inflammatory gene expression, it is extremely important to have a robust model that predicts kinetic of NF-κB phosphorylation. Therefore, we aimed to describe dose response and kinetics of the IKK mediated phosphorylation of NF-κB p65 subunit. Previous findings enabled us to refine and construct a new model combining both NF-κB phosphorylation and translocation processes and predict the existence of a negative feedback loop inactivating phosphorylated NF-κB. To our knowledge, we present the first model that accounts for the NF-κB phosphorylation process in NF-κB nuclear translocation. This could be a starting point to further extend NF-κB signaling models considering changes in phosphorylation-dependent protein interactions and gene induction.

## Results

### LPS Induces fast activation of the NF-κB pathway in primary macrophages

We studied the kinetics of LPS-induced NF-κB-activation in primary BMMϕ using both flow cytometry and confocal imaging. Due to the heterogeneity of GM-CSF-grown macrophages^20^, we accurately monitored the purity of cells in all of our experiments, maintaining a purity level of at least 95% (Fig. S1). It is well-known that within a few hours of TLR4 activation, macrophages begin secreting TNFα, IL-1β and other cytokines^4^. To avoid any possible effect of autocrine and paracrine cytokines on NF-κB signaling^17^, we focused on the early signaling events occurring within the first two hours.

**Figure 1:**
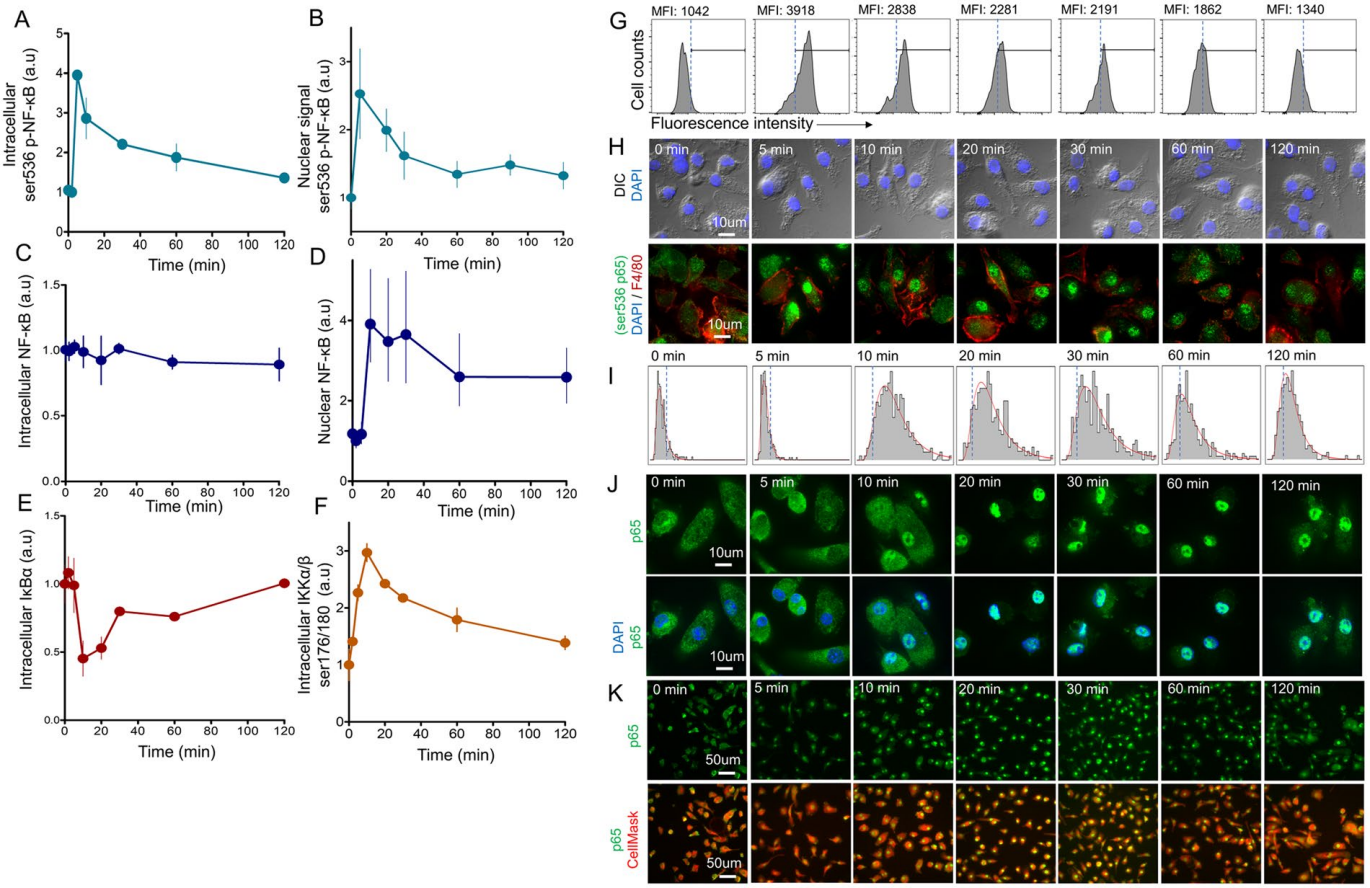
Kinetics of NF-κB activation events within LPS-stimulated bone marrow derived macrophages. (**A**) Kinetics of NF-κB phosphorylation (ser 536, p65) measured by flow cytometry (n = 4 replicates) at LPS concentration of 500 ng/ml. (**B**) The nuclear mean signal of phosphorylated p65 subunit of NF-κB (ser 536 p65), measured by confocal microscopy. (**C**) Kinetics of the overall level of intracellular NF-κB (p65) measured by flow cytometry (n = 2 replicates). (**D**) Kinetics of NF-κB nuclear translocation measured by confocal microscopy. (**E**) Kinetics of IκBα degradation measured by flow cytometry (n = 3 replicates). (**F**) Kinetics of IKKα/β phosphorylation (ser 176/180) measured by flow cytometry (n = 4 replicates). All replicates are biological. (**G**) Representative flow cytometry histograms of cells stained with antibodies recognizing the phosphorylated form of NF-κB (ser 536), taken at different time points. (**H**) Confocal images of NF-κB phosphorylation kinetics (ser 536 p65). Cells stained with antibodies recognizing phosphorylated NF-κB (green), macrophages surface marker F4/80 (red), and DAPI (blue). (**I**) Histograms of NF-κB(p65) nuclear signal distribution over time measured in macrophages via confocal microscopy. (**J**,**K**) Confocal images of NF-κB (p65) nuclear translocation kinetics. Cells stained with antibodies to the p65 subunit of NF-κB (green), CellMask^TM^ Deep Red (cytosol, red), and DAPI (blue) and captured at x100 (**J**), x20 (**K**). Flow cytometry data are presented as mean ± SD normalized to unstimulated cells. Confocal data are presented as median ± 25% and 75% quantiles of signal distribution within cells normalized to control values.

Activation of TLR4-dependent signaling processes in macrophages was estimated by various methods, in particular IKKα/β phosphorylation, IκBα degradation and NF-κB phosphorylation kinetics, as well as nuclear translocation of both phosphorylated and non-phosphorylated NF-κB (Fig. 1). At different time points following activation, BMMϕ were fixed, permeabilized and labeled with fluorescent antibodies, after which intracellular levels of IκBα and phosphorylated NF-κB were measured using flow cytometry. In this way, we recorded the total cellular levels of IκBα and phosphorylated proteins. Fig. 1A represents experimental NF-κB phosphorylation dynamics (serine 536 phosphorylated p65 subunit of NF-κB) measured after the addition of LPS to the culture of macrophage. After a 1–2 min delay the phosphorylation signal rapidly increased, reaching its’ maximum at 5 min, then slowly decreasing to the initial values over the next 2 hours. The kinetics of phosphorylated NF-κB translocation from the macrophage cytoplasm to nucleus is shown in Fig. 1B,H. It coincided with the shape of total cell NF-κB phosphorylation kinetics (Fig. 1A). Maximal nuclear-phosphorylated NF-κB was also achieved 5 min after LPS stimulation. Moreover, according to the confocal images shown in Fig. 1H, the kinetics of cellular NF-κB phosphorylation were almost completely defined by the nuclear fraction. This is probably due to a very high rate of the phosphorylated NF-κB nuclear translocation, causing the phosphorylated NF-κB to be detected predominantly in the nucleus of LPS-activated macrophages.

While the total amount of intracellular NF-κB (cytoplasmic and nuclear) remained unchanged (Fig. 1C), the kinetics of NF-κB translocation into the cell nucleus were similar to its’ phosphorylation dynamics, though slightly slower (Fig. 1D). NF-κB translocation into the nucleus was determined by confocal microscopy using the method described in Trask *et al*. (2011). At the appropriate time points cells were immediately fixed and stained with antibodies. Focal location of the cell nucleus was defined with DAPI, and the mean nuclear NF-κB fluorescence intensity was measured (example of kinetic images shown in Fig. 1J,K). The nuclear NF-κB level reached its’ maximum 10 min after the addition of LPS, then gradually decreased to a half-maximal level over the following 100 min, reflecting the IκBα degradation kinetics (Fig. 1E). Within 10–20 min of activation by LPS, the IκBα concentration in macrophages rapidly dropped to its’ minimum before slowly returning back to baseline (pre-activation).

NF-kB distribution within nuclei of activated macrophages had uni-modal log-normal distribution (Figs 1I and S2) (Cramer-Von Mises criteria p-value for log-normal distribution p = 0.67). We did not detect significant deviation from single modal distribution and existence of additional peaks within distribution during the observation period (Fig. S2). Analysis of NF-kB kinetics via confocal microscopy did not show that population of macrophages heterogeneously react on the LPS stimulation.

### An adjusted mathematical model describing TLR4-induced activation of NF-κB in primary BMMϕ explains rapid NF-κB activation

**Figure 2:**
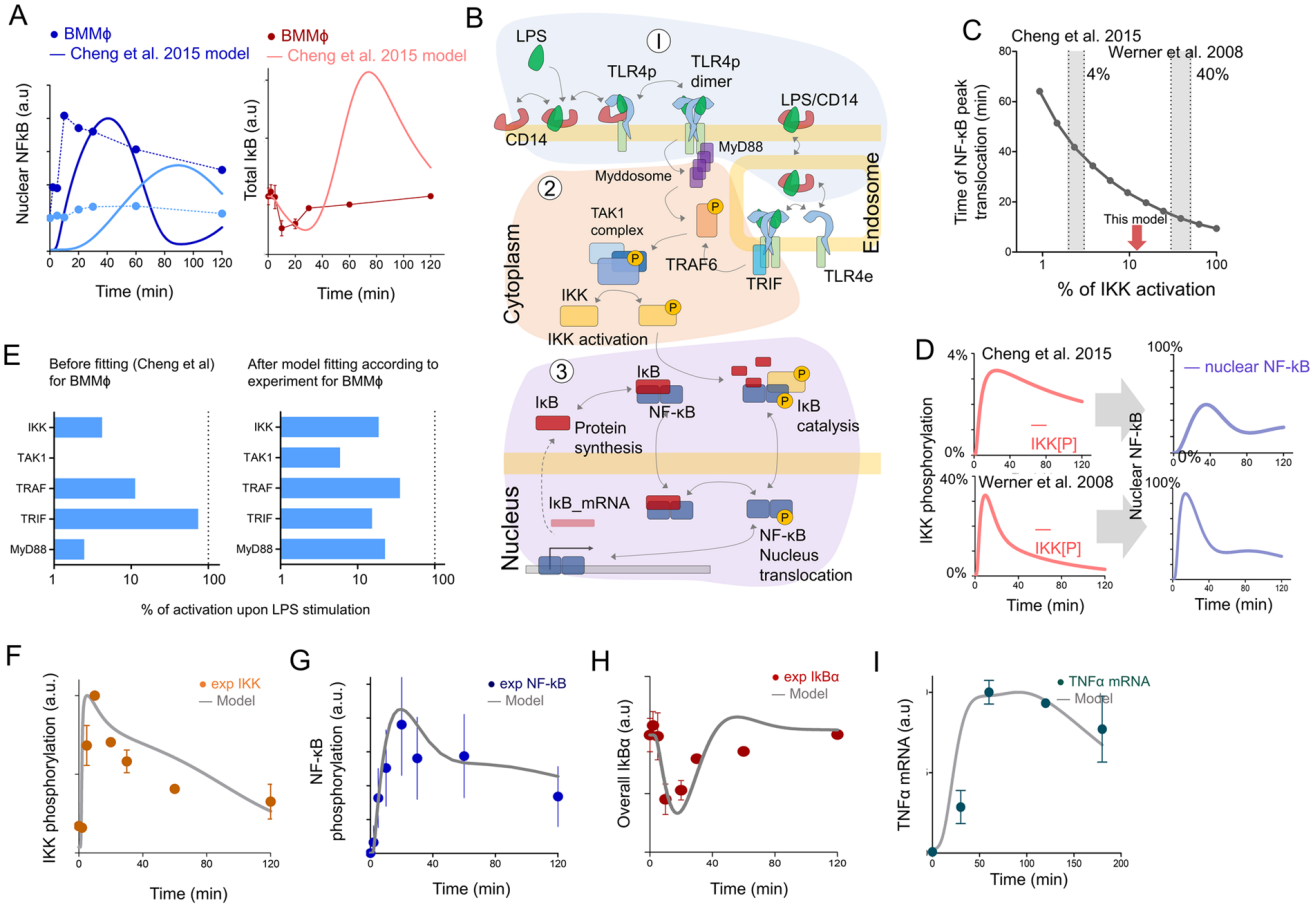
Updating existing mathematical models to correctly describe NF-κB translocation kinetics in primary BMMϕ. (**A**) Comparison of NF-κB translocation kinetics (upon 500 and 1 ng/ml LPS stimulation) of primary BMMϕ (dots) and IkB degradation kinetics (dots) in our experiments with calculations derived from the Cheng *et al*. 2015 model^8^. (**B**) Schematic representation of TLR4 signaling cascade used for mathematical modeling. The signaling was divided into 3 modules: (1) TLR4/LPS binding module, (2) IKK activation module, (3) NF-κB activation and translocation. For More information see S1 Methods. (**C**) *In silico* calculated dependence of NF-κB translocation peak time on the amount of IKK phosphorylation (percent from IKK maximum activation). Shaded areas correspond to IKK activation levels in Cheng *et al*.^8^ and Werner *et al*.^73^ models of NF-κB signaling. Cheng *et al*. (2015) described maximum IKK activation at 4%, where the minimum time to first peak was 30–40 min for RAW cells. Werner *et al*. (2008) described IKK activation up to 40% of maximum, achieving a peak before 10–15 min (MEF cells). Red arrow showed model predicted values. (**D**) Model calculation of NF-κB nuclear translocation at different IKK kinetic shapes. The examples of IKK kinetic shapes are collected from Cheng *et al*. (2015) and Werner *et al*. (2008). (**E**) Left - Maximum activation of intermediate species in the IKK-module (IKK, TAK1, TRAF6, TRIF, Myd88) before refitting of kinetic parameters in the Cheng *et al*. (2015) model for BMMϕ, right – Maximum activation of intermediate species after refitting of kinetic parameters. (**F–I**) Comparison of model calculations (solid lines) with experimental data (dots) after refitting of parameters. Dynamics of IKKα/β (ser 176/180) phosphorylation (**F**), total IκBα (**H**), NF-κB (p65) translocation (**G**), and TNFα mRNA synthesis (normalized on β-actin) (**I**) measured at LPS concentration of 500 ng/ml and presented in relative units.

The rate of NF-κB translocation and its’ kinetics shape seems to be strongly variable across different cell types. For example, Covert *et al*. (2005) showed that the NF-κB nuclear translocation in MEFs achieved its’ maximum at 80–100 min. The peak NF-κB nuclear translocation for 3T3 and RAW 264.7 cells has been recorded at 50 min and 30 min, respectively^8,14^ (Fig. S3). For bone marrow macrophages, Cheng *et al*. (2015) measured a 20 min peak time, similar to our experimental data, and introduced the most comprehensive mathematical model of TLR4 signaling, mostly tuned for RAW 264.7 macrophage cell line. However, provided mathematical model failed to account for our experimental data (Fig. 2A).

The main problems were connected with two issues:

- Existing models could not describe the fast kinetics of NF-κB nuclear translocation in response to various LPS concentrations than observed in the experiment.
- The model kinetics of intermediate species like IκBα or phosphorylated IKK was not in concordance with experimental measurements for primary macrophages

We therefore constructed a model describing both TLR4-induced NF-κB phosphorylation and translocation into the nucleus in primary BMMϕ. Our model is a serious extension of^13^ and^8^ (S1 Methods, S4 Table, Figs 7–10 especially for the case of NF-κB phosphorylation. We divided the TLR4 pathway into three modules (Fig. 2B). Module 1, or the TLR4 module, describes the interaction of LPS with the TLR4 receptor, the receptor dimerization and following endocytosis of the receptor-ligand complex. Module 2, or the IKK module, comprises signal transduction through the TRAF6/TAK1 complexes to the endpoint IKK complex (NEMO/IKKα/β) phosphorylation. Module 3, or the NF-κB module, is a block of reactions describing IKK-dependent catalysis of IκB, the NF-κB inhibitor, and the negative feedback loop connected with induction of IκB resynthesis.

At first our goal was to explain the observed NF-κB translocation and IkB degradation kinetics in BMMϕ which could not be fitted with existing models; the maximum NF-κB translocation peak should be obtained 10 min after LPS stimulation. With that said, and assuming that all equations proposed by other models were correct, we had to refit some values of the kinetic parameters. We analysed sensitivity for each model parameter that could influence the dynamics of NF-κB translocation (Fig. S4). The parameters of the IKK activation module (module 2, TRAF6 activation, TAK1 activation and IKK phosphorylation) were found to be the most sensitive for NF-κB translocation. Many of these parameters were defined by Cheng *et al*. (2015) by fitting for the RAW 264.7 macrophage cell line and partially for BMMϕ^8^. We refitted only parameters of high sensitivity previously defined not for BMMϕ. Such calculations revealed that the concentration of activated IKK and its’ kinetics are some of the most important regulators of the NF-κB translocation rate (Fig. 2C). For example, in the similar system of NF-κB activation by TNFα receptor signaling, Werner *et al*.^22^ used fast IKK phosphorylation at 30% of the theoretical maximum, thus achieving an NF-κB translocation peak at 10 min (Fig. 2D). By comparison, Cheng’s model delayed the maximum peak time position to 40 min with IKK activation strength at 3% of the possible maximum. Furthermore, shape of NF-κB kinetics are mostly determined by the shape of IKK deactivation kinetics, which could be sharp and rapidly decreasing, as in Werner *et al*., or slow, as in Cheng *et al*. resulting in the corresponding shape of NF-κB kinetics (Fig. 2D).

We therefore refined the parameters of the IKK-module activation for TAK1, TRAF6, TRIF and MyD88, such that activated species had biologically relevant values (Fig. 2E). The maximum IKK activation after fitting was changed from 0.003 µM, which corresponds to 3% of the maximum value, to 0.02 µM, corresponding to 20% of the maximum value (Fig. 2E). For high LPS concentrations, this brought the model kinetics of phosphorylated IKK into agreement with our experimental data in BMMϕ and allowed us to adjust NF-κB kinetics peaks to 10–20 min. This allowed us to better describe the experimental results of NF-κB translocation and IKK activation kinetics (Fig. 2F,G).

The main difficulties we encountered were with the realization of equations describing inducible IκB mRNA synthesis^8^. Previous models used an assumption that when NF-κB enters the nucleus it initiates transcription by its own, leaving aside a complicated processes of NF-κB binding to DNA and polymerase complex formation^13,23^. Other authors implemented an artificial delay in mRNA transcription relative to the NF-κB nuclear translocation kinetics^6,8,24^ not supported with ODE equations. We used a direct reactions of NF-κB / DNA complex formation. Although the NF-κB-DNA binding kinetics is very fast^25–27^, in-depth analysis showed that such process can provide the intended delay in the induction of gene transcription. Direct NF-κB binding to DNA let us better describe synthesis of IκBα and even mRNA synthesis of NF-κB target genes like TNFα without functions of artificial delay (Fig. 2H,I).

### Mathematical calculations predicted that preexisting basal nuclear NF-κB levels could accelerate NF-κB translocation kinetics at low concentrations of TLR4 agonist

**Figure 3:**
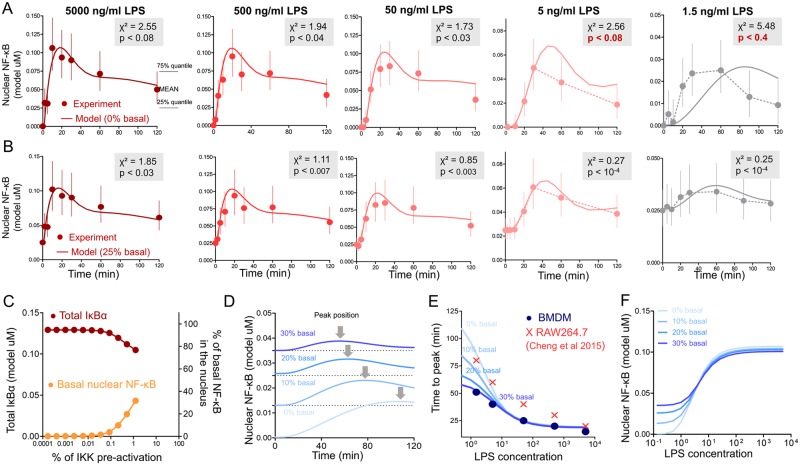
Influence of basal NF-κB levels on NF-κB translocation kinetics. (**A**) Fitting of experimental NF-κB nuclear translocation data measured by confocal microscopy in unstimulated BMMϕ to the model with initial 0% basal nuclear levels of NF-κB. (**B**) Fitting of experimental NF-κB nuclear translocation data measured by confocal microscopy in unstimulated BMMϕ to the model with 25% basal level NF-κB. Experimental data in A and B (dots) presented as median ± 25% and 75% quantiles of signal distribution within cells, data obtained from more than 500 cells per time point, from 4 biological replicates. Chi-square goodness-of-fit test is presented in the upper right corner. To achieve zero NF-κB at time 0 for experimental dots, the value of nuclear NF-κB signal before LPS activation was subtracted. (**C**) The calculated steady state dependence of total IκBα concentration (left axis) and percent of basal nuclear NF-κB (percent from total NF-κB) on the IKK pre-activation level (percent of phosphorylated IKK from total IKK). (**D**) Predicted changes in the kinetics of NF-κB translocation upon stimulation with low concentration LPS (1.5 ng/ml) calculated for varying basal NF-κB levels (0, 10, 20, 30%). (**E**) Model predictions (solid lines) of time to peak NF-κB related to LPS concentration, calculated for 0, 10, 20 and 30% of initial basal NF-κB levels. Experimental data (dots) of time to peak NF-κB translocation for BMMϕ (●), and according to Cheng *et al*. (2015) in RAW 264.7 cell line (**x**). (**F**) Dependence of NF-κB translocation amplitude on LPS concentration calculated for different basal NF-κB levels (0, 10, 20, 30%).

We observed that NF-κB nuclear translocation kinetics were remarkably dependent on the initial concentration of TLR4 (Fig. 3). Specifically, a decrease in the LPS concentration from 50 to 2 ng/ml led to a threefold increase in the time taken to reach maximal nuclear NF-κB levels (Fig. 3A). In addition to this increase, the amplitude of NF-κB dynamics was also dependent on LPS concentration. Therefore, we were most interested in the model’s precise prediction of the systematic response to a wide range of LPS concentrations.

While the updated model of NF-κB activation and translocation kinetics at high LPS concentrations perfectly matched our experimental data (Fig. 2F–I), at low LPS concentrations (1–5 ng/ml), the model poorly described observed curves (Fig. 3A). Almost all kinetic parameters (primarily from modules 1 and 2, Fig. 2A) were varied to find the best fit without additional model changes, but this approach was unsuccessful.

During postprocessing of confocal images or western blots of NF-κB translocation the nuclear NF-κB signal from unstimulated cells is usually treated as a background relative to which the overall kinetics is measured. However we hypothesized that background or basal level of NF-κB, observed in the nucleus of non-activated BMMϕ (Fig. 1I) should be included in the overall NF-κB translocation kinetics.

To describe the appearance of basal amounts of NF-κB in the nucleus prior to TLR4 activation in the model, we hypothesized existence of an unknown process that produces IKK phosphorylation at a constitutive level (Fig. S9, S1 Methods). Only the IKK complex able to triggers degradation of IκB proteins and allows translocation of NF-κB in the nucleus of macrophages without external signals. Stable state of the system with increased basal level of activated IKK induce low rate degradation of IκBα and generation of basal NF-kB translocated in the nucleus (Fig. 3C). The rate of IκBα / NF-κB interaction in the cell so high, so that free NF-κB could not exist with overrepresented IκBα.

Model analysis revealed that the NF-κB nuclear translocation rate could be regulated by the basal NF-κB pre-activation. According to the model, the time to peak NF-κB translocation into the nucleus depends on the basal NF-κB level (Fig. 3D,E). At zero basal nuclear NF-κB (0%) and a low initiating concentration of LPS (1 ng/ml), the maximum response is reached within 110 min. A 10% basal nuclear level of total cellular NF-κB, decreases the the NF-κB translocation peak to 75 min (Fig. 3D). A basal nuclear level of 30% reduces this time twofold to 55 min.

The addition of the high basal pre-activated NF-κB to the model allowed us to better explain the experimental curves of the NF-κB translocation kinetics in the BMMϕ (Fig. 3B). If we assume that 25% of all NF-κB is already located in the nucleus prior to TLR4 activation, the model better describes experiments with low concentrations of LPS (5 ng/ml to 1.5 ng/ml), decreasing chi-square fit p-value from 0.4 to 10^−4^ (Fig. 3B).

We laid out time to peak NF-κB translocation for macrophages measured under our experimental conditions on the in silico calculated dependence for different basal conditions. In the case of bone marrow macrophages, the best matching of model calculations and experimental data was achieved at 30% of the basal NF-κB level (Fig. 3E). It is interesting that the zero basal level should had been and was observed in RAW macrophages from the work of Cheng *et al*., on which our model was based (Fig. 3E). Model also predicted that above LPS concentrations of 50 ng/ml the time to maximal response does not depend on basal NF-κB level (Fig. 3E). And the preexisting basal nuclear NF-κB level weakly influences the TLR4 activation threshold, keeping it almost unchanged (Fig. 3F).

**Figure 4:**
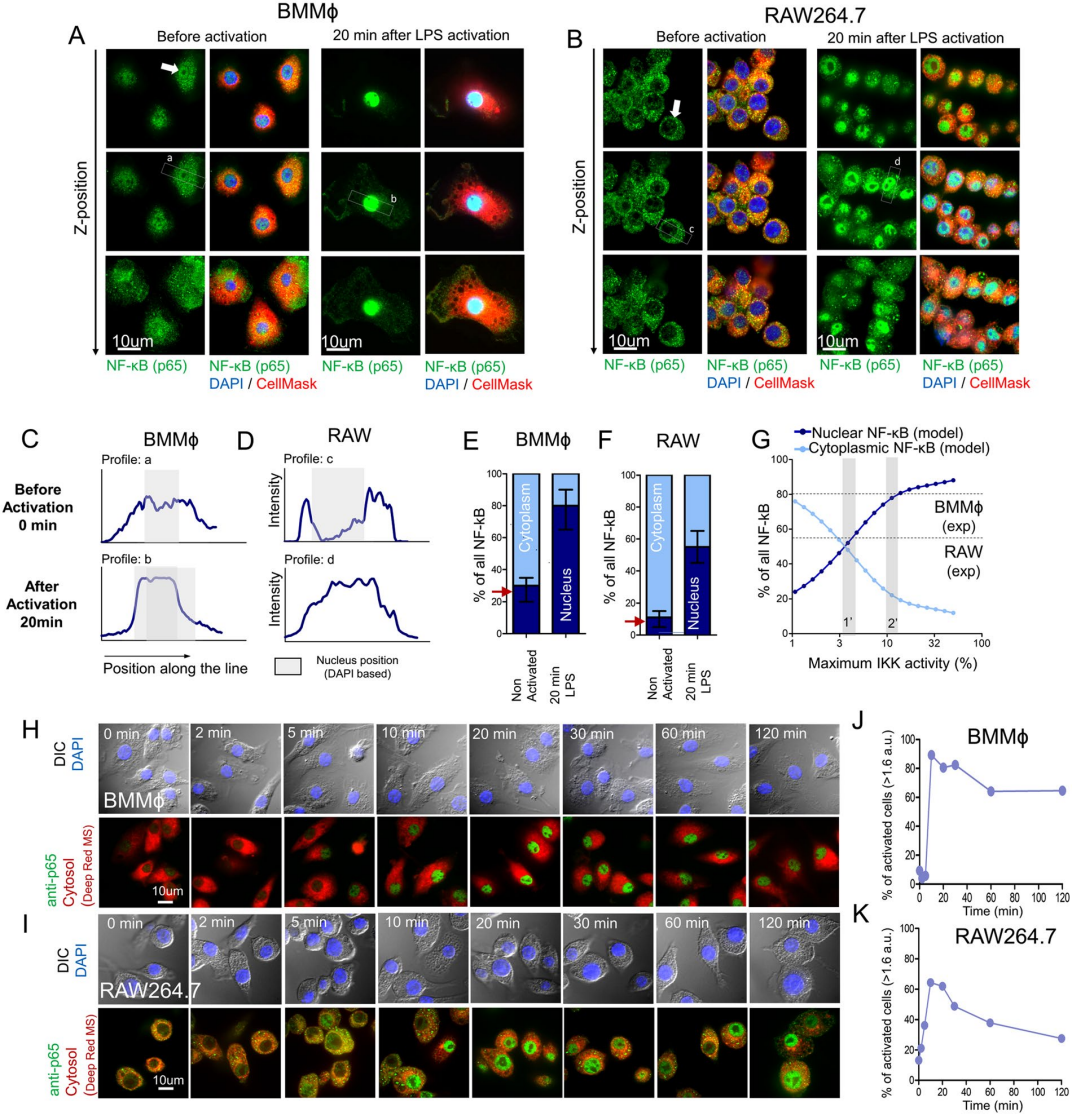
Basal NF-κB Levels in RAW 264.7 Macrophages and Bone Marrow-Derived Macrophages. Z-stack confocal analysis of NF-κB distribution in BMMϕ (**A**) and RAW 264.7 cells (**B**) before (left) and 20 min after (right) LPS stimulation (500 ng/ml); 3 different confocal positions on the Z axis are presented. Cells were stained with anti-p65 antibodies (green) and counterstained with CellMask^TM^ Deep Red (cytosol, red) and DAPI (blue). Profile plot of NF-κB fluorescence signal in BMMϕ (**C**) and RAW 264.7 cells (**D**) before and 20 min after LPS activation measured along a,b,c and d lines depicted on the Figures A,B. (**E**,**F**) Intracellular distribution of NF-κB between the nucleus and cytoplasm before and 20 min after LPS activation in RAW (**E**) and BMMϕ cells (**F**) measured by confocal microscopy. Red arrows represent model predicted values. (**G**) Dependence of intracellular NF-κB distribution on maximum IKK activation predicted by the model. Shaded areas correspond to maximum IKK activation in Cheng *et al*. (2015) model (1′) for RAW 264.7 cells and previously predicted by model (2′) for BMMϕ (Fig. 3C,E). (**H–I**) Confocal images of NF-κB (p65) nuclear translocation kinetics in BMMϕ cells (**H**) and RAW264.7 cells (**I**). Cells stained with antibodies to the p65 subunit of NF-κB (green), CellMaskTM Deep Red (cytosol, red), and DAPI (blue) and captured at x100. (**J–K**) Percent of activated cells measured as a percent of cells with nuclear NF-κB intensity above 1.6 a.u. (see Fig. 1F) in BMMϕ cells (**J**) and RAW264.7 cells (**K**)

Our further studies revealed that indeed BMMϕ had a higher basal nuclear NF-κB level as compared to RAW 264.7 macrophages (Fig. 4). We, therefore, performed Z-stack confocal imaging for reconstruction of NF-κB location in cell compartments. This approach showed that unstimulated primary macrophages had approximately 30% of total cellular NF-κB in their nuclei, similar to values predicted by our model (Fig. 4A,C,E). Without any activation, the vast majority of primary macrophages labeled with a fluorescent antibody to NF-κB, had a higher nuclear fluorescence intensity than cytoplasmic fluorescence intensity, which can be easily seen at the profile plot at Fig. 4C. Twenty minutes after activation of BMMϕ with LPS, about 80–90% of the total cellular NF-κB localized in the nucleus (Fig. 4E). Thus, high pre-activation (basal) levels of nuclear NF-κB, were observed in primary BMMϕ in our study, but nearly zero pre-activation levels of nuclear NF-κB were reported in previous experiments and mathematical models on transformed macrophage cell lines^8,28^. Although, the naive bone marrow derived macrophages did show increased basal NF-κB in the similar conditions^29^.

As well as in other studies, we found that the basal nuclear NF-κB level in RAW 264.7 macrophages was rather low, at no more than 5–10% of total cellular NF-κB (Fig. 4B,D). Following LPS stimulation, levels of nuclear NF-κB in RAW 264.7 reached 50–60% of total NF-κB (Fig. 4F). It is important to note that NF-κB translocation to the nucleus is also dependent on IKK activation strength. The model calculation showed that IKK must be activated to at least 20% (Fig. 4G) in order to achieve 80–90% of all NF-κB translocated into the nucleus (as observed in BMMϕ). That’s matches previously fitted values for maximum IKK phosphorylation strength for BMMϕ (Fig. 2C–E).

In contrast to RAW264.7 cells, native macrophages simultaneously reacted on the LPS stimulation. Increase and decrease of NF-κB nuclear signal in macrophage cells occur simultaneously (Fig. 4H). The percent of activated BMMϕ remained high during the period of observation (Fig. 4J). In the same conditions the snapshots of NF-κB translocation in the RAW264.7 cells did contain “switched off” cells after 1 hour of observation (Fig. 4I), thus having a lowered percent of activated cells and possible oscillations of NF-κB (Fig. 4K). Which evidenced for more homogeneous and synchronized NF-kB nuclear translocation response in native macrophages in comparison to RAW264.7 cell line.

We carefully maintained all culture conditions the same for all the cell types while performing our experiments. Even co-cultivation of mixed BMMϕ and RAW264.7 cell populations within the same culture did not change the NF-kB levels in their nuclei. In such co-cultures, RAW264.7 cells firmly kept their nuclei empty of NF-kB while BMMϕ still had high basal NF-kB level in their nuclei (Fig. S5A). And these findings were not associated with antibody clone (Fig. S6).

### Experimental measurement of NF-κB nuclear translocation kinetics in three types of macrophages with different basal levels of nuclear NF-κB

**Figure 5:**
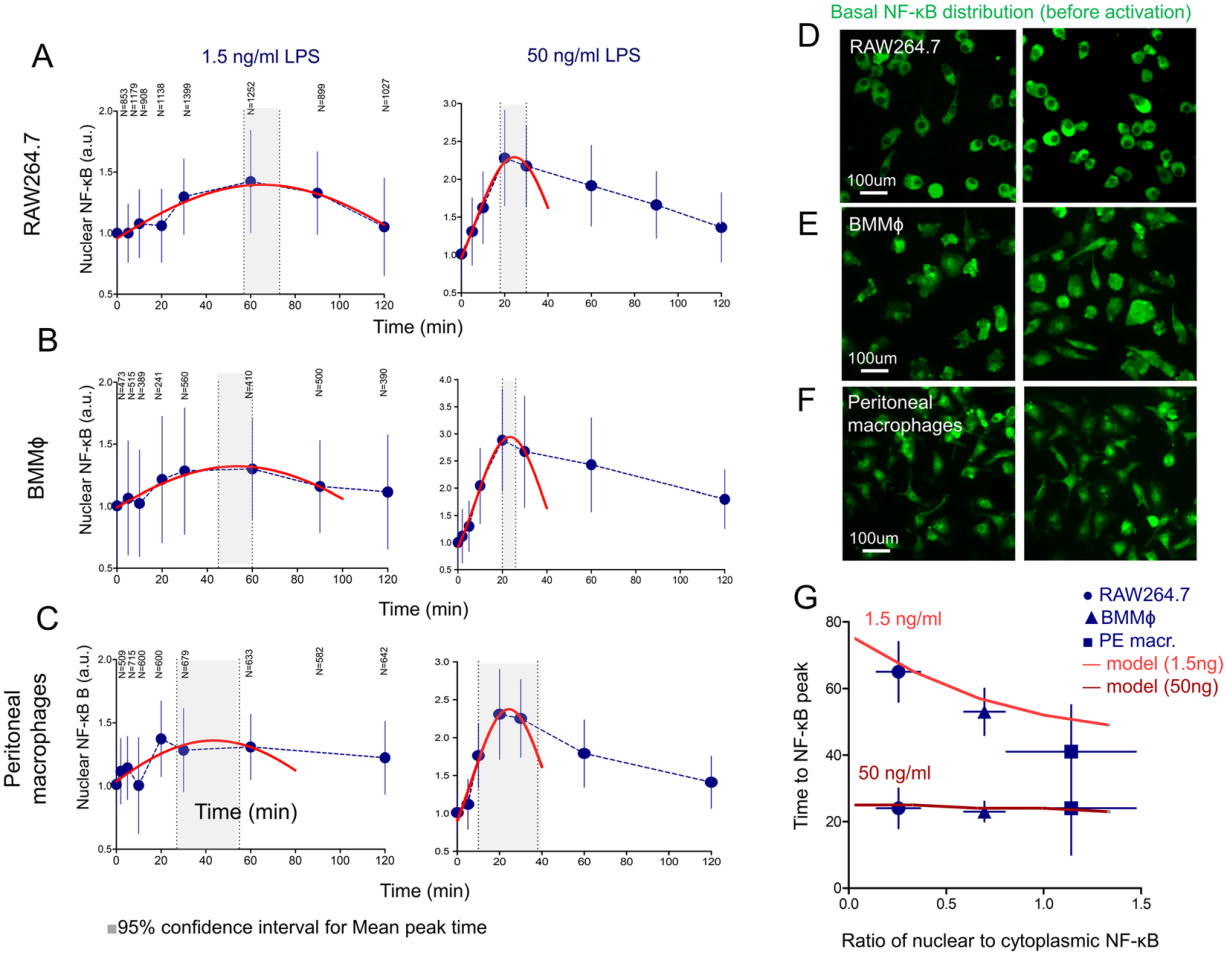
The difference between RAW 264.7 cells, bone marrow-derived and peritoneal macrophages in relation to NF-κB translocation kinetics after low-concentration LPS stimulation. (**A**,**B**,**C**) NF-κB translocation kinetics in RAW 264.7 cells (**A**), BMMϕ (**B**) and peritoneal macrophages (**C**) measured by confocal microscopy at 1.5 ng/ml of LPS (left) and 50 ng/ml of LPS (right) concentrations. Shaded areas are 95% confidence intervals for the peak NF-κB position, calculated with Gauss non-linear fit of experimental data. Red lines show the Gauss fit. N values represent the number of cells analysed at each time point. Experimental data (dots) are presented as median ± 25% and 75% quantiles. (**D**–**F**) Representation of the basal NF-κB distribution in unstimulated RAW 264.7 cells (**D**), BMMϕ (**E**) and peritoneal exudate macrophages (**F**). (**G**) Dependence of time to NF-κB peak position on basal ratio of nuclear to cytoplasmic NF-κB for 1.5 and 50 ng/ml of LPS activation, experiment (dots) and model (lines). Different basal ratios obtained from different cell type (●RAW264.7, ▲BMMϕ, ■ peritoneal macrophages), mean peak time positions obtained from gauss fit of (**A**–**C**). Experimental data (dots) are presented as mean ± SD, ±95% confidence interval for peak time.

We compared the kinetics of NF-κB nuclear translocation in 3 different cultures of macrophages: primary BMMϕ, primary peritoneal exudate macrophages and RAW 264.7 cell line macrophages (Fig. 5). Mouse peritoneal macrophages, isolated immediately prior to the experiment, had the highest basal NF-κB level (Fig. 5F) as compared to BMMϕ (Fig. 5E) and RAW 264.7 cells (Fig. 5D). Upon activation with low-dose LPS (1.5 ng/ml), the kinetics of NF-κB nuclear translocation were faster in primary macrophages than RAW 264.7 (Fig. 5A–C). The most probable position of the first peak was approximated using the Gauss fit of experimental data. Upon activation with a low concentration of LPS, NF-κB translocation kinetics in RAW 264.7 macrophages peaked at 70 min (95% confidence interval 57–74 min), at 52 min (40–60 min) in BMMϕ and at 41 min (27–54 min) in peritoneal macrophages (Fig. 5A–C, left). This was found to be in fair agreement with our model, which predicted decrease of time to peak in dependence of basal NF-κB level (Fig. 5G, top line). Peritoneal macrophages had the highest basal NF-κB level in comparison with other macrophages and had the fastest time response at 1.5 ng/ml of LPS. At the higher TLR4 agonist concentration (50 ng/ml), there was no difference between the primary and transformed macrophages, which was in good agreement with our model’s predictions as well (Fig. 5G, bottom line).

### A precise description of NF-κB phosphorylation kinetics predicts the existence of a negative regulation that is different from the A20-like deubiquitination mechanisms

There are numerous gene promoters regulated by NF-κB^2,30,31^. It is believed that site-specific phosphorylation determines which genes are directly targeted by phosphorylated NF-κB^32–34^. It is therefore important to understand whether NF-κB phosphorylation could be described within the NF-κB translocation model. The IKKα/β complex is thought to be responsible for NF-κB phosphorylation at serine 536 residue^19,34^. It is reasonable to presume that phosphorylation happens upon IKK binding to the IκB/NF-κB complex, which is the only time when NF-κB can be exposed to IKK in the cytoplasm. Upon degradation of IκB, NF-κB immediately translocates to the nucleus^12^. Model calculations confirm that almost all detected phosphorylated NF-κB should be located in the nucleus, which is in accordance with our experimental data (Fig. 1B). Due to rapid NF-κB translocation, the IKK-induced NF-κB phosphorylation rate must be relatively high.

**Figure 6:**
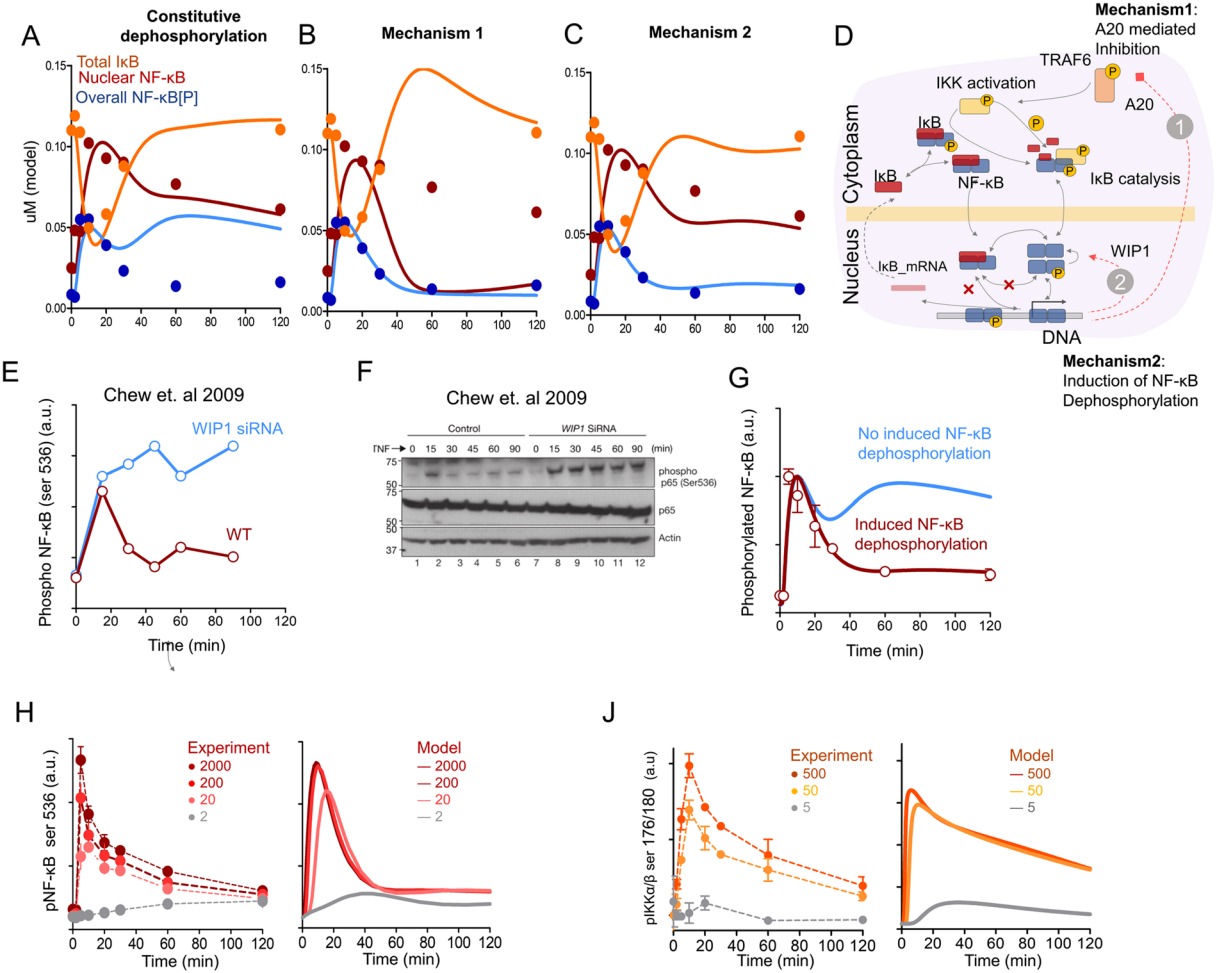
Model description of NF-κB phosphorylation kinetics. (**A**) Kinetics of nuclear, phospho-NF-κB and total IκB calculated in the model (line) in the system of constitutive de-phosphorylation of NF-κB in the nucleus. Overall amount of phosphorylated NF-κB[P] (light blue), overall nuclear NF-κB (red line), total IκB (orange line). Corresponding experimental mean values are presented as dots. (**B**) Kinetics of nuclear, phospho-NF-κB and total IκB calculated in the model (line) to fit NF-κB phosphorylation kinetics by A20-related inhibition. Corresponding experimental mean values are presented as dots. (**C**) The same as in (**B**) except fitted with NF-κB dephosphorylation induced by negative feedback loop via WIP1. (**D**) Schematic description of NF-κB phosphorylation and nuclear translocation processes described in the model. Two possible mechanisms for the negative regulation of NF-κB phosphorylation are presented: 1 – Induction of A20 synthesis, which opposes TRAF6-dependent ubiquitination processes; 2 – Induction of a factor that directly dephosphorylate NF-κB (possibly via WIP1). (**E**,**F**) Kinetics of NF-κB phosphorylation (ser 536) after TNF stimulation of HeLa cells without (red line) or with (blue line) inhibition of WIP1 mRNA synthesis with siRNA. Data derived from Chew *et al*. 2009. The initial western blot (**F**) and the reconstructed curves (**E**) are shown. (**G**) Blue line - a model curve of phosphorylated NF-κB kinetics in the proposition of constitutive dephosphorylation in the nucleus plus IκBα-mediated dephosphorylation. Red line - a model curve under proposed conditions of induced dephosphorylation of NF-κB through WIP1, as described in the article. (Dots) Experimental data of phosphorylated NF-κB kinetics (ser 536, p65) in BMMϕ activated with 500 ng/ml of LPS. (**H**) NF-κB (ser 536-p65) phosphorylation kinetics at different LPS concentrations (from 2 to 2000 ng/ml) in BMMϕ. Experimental flow cytometry data (n = 4 biological replicates) (dots, left) and model (lines, right) presented in normalized units. (**J**) IKKα/β (ser 176/180) phosphorylation kinetics at different LPS concentrations (from 5 to 500 ng/ml) in BMMϕ. Experimental flow cytometry data (n = 2 biological replicates) (dots, left) and model prediction (lines, right) presented in normalized units.

Two hours after stimulation more than 50% of all NF-κB still remains in the nucleus (Fig. 1D), and IKK activity remains high (Fig. 1F), but the phosphorylated form of NF-κB disappears (Fig. 1A). It is logical to assume that dephosphorylation of NF-κB occurs in the nucleus^15,19^. This could possibly explain the sharp experimental kinetics of phosphorylated NF-κB with maximum achieved at 5 min, followed by a quick decrease. However, constitutive dephosphorylation of NF-κB alone could not explain observed phosphorylation dynamics (Fig. 6). In this case, the model predicts that activated NF-κB enters the nucleus from the cytoplasm, induces synthesis of IκBα, then returns to the cytoplasm^26^, is phosphorylated by IKK and returns back to the nucleus, creating oscillations or a constant level of phosphorylated NF-κB as predicted with the model) with constitutive dephosphorylation (Fig. 6A, blue line).

It seems that, for the concordance of the experimental data and the model, one more important negative feedback mechanism induced by LPS activation is necessary to regulate NF-κB phosphorylation kinetics (Fig. 6D). The most well-known tolerogenic negative feedback loop in the NF-κB system is the synthesis of A20-like proteins that deactivate the upstream signaling^35^ (Fig. 6B,D). It is known, for example, that A20 deubiquitinase interferes with the TRAF6-induced ubiquitination processes^36^. However, in order to fit experimental NF-κB phosphorylation kinetics with A20 related mechanisms, the overall NF-κB translocation kinetics must be deactivated at 60 min post-LPS stimulation, which is not observed in the experimental data (Fig. 6B).

If we assume existence of more delicate regulation of NF-kB phosphorylation via induction of certain de-phosphatases we could explain the observed kinetics of all species (Fig. 6C). Certain proteins, for example C/EBP^37^, PP2A^38^, PP1^39^ or WIP1^40^ are associated with induced p65 dephosphorylation. For example, inhibition of synthesis of WIP1 phosphatase creates a constant level of phosphorylated NF-κB^40^ (Fig. 6E,F, image derived from Chew *et al*.). The model predicts similar constant level of phosphorylated NF-kB without induced dephosphorylation of NF-kB (Fig. 6G, blue line). We included in the model the NF-κB inducible WIP1-dependent dephosphorylation process which allowed us to accurately describe the shape of NF-κB phosphorylation kinetics (Fig. 6G, red line) and explain the experiments of Chew *et al*.^41^. Addition of WIP1 induced dephosphorylation enabled to explain changes of phospho-NF-kB (ser 536) kinetics in dependence on LPS concentration in relation to the IKK activity (Fig. 6H). We observed a fast peak at 5–10 min followed by fast decrease of total NF-κB phosphorylation (Fig. 6H), while phospho-IKKα/β kinetics appeared to be more elongated in time (Fig. 6J).

### The updated model accurately describes a shift of the amplitude and peak time for NF-κB activation kinetics, depending on LPS concentration

**Figure 7:**
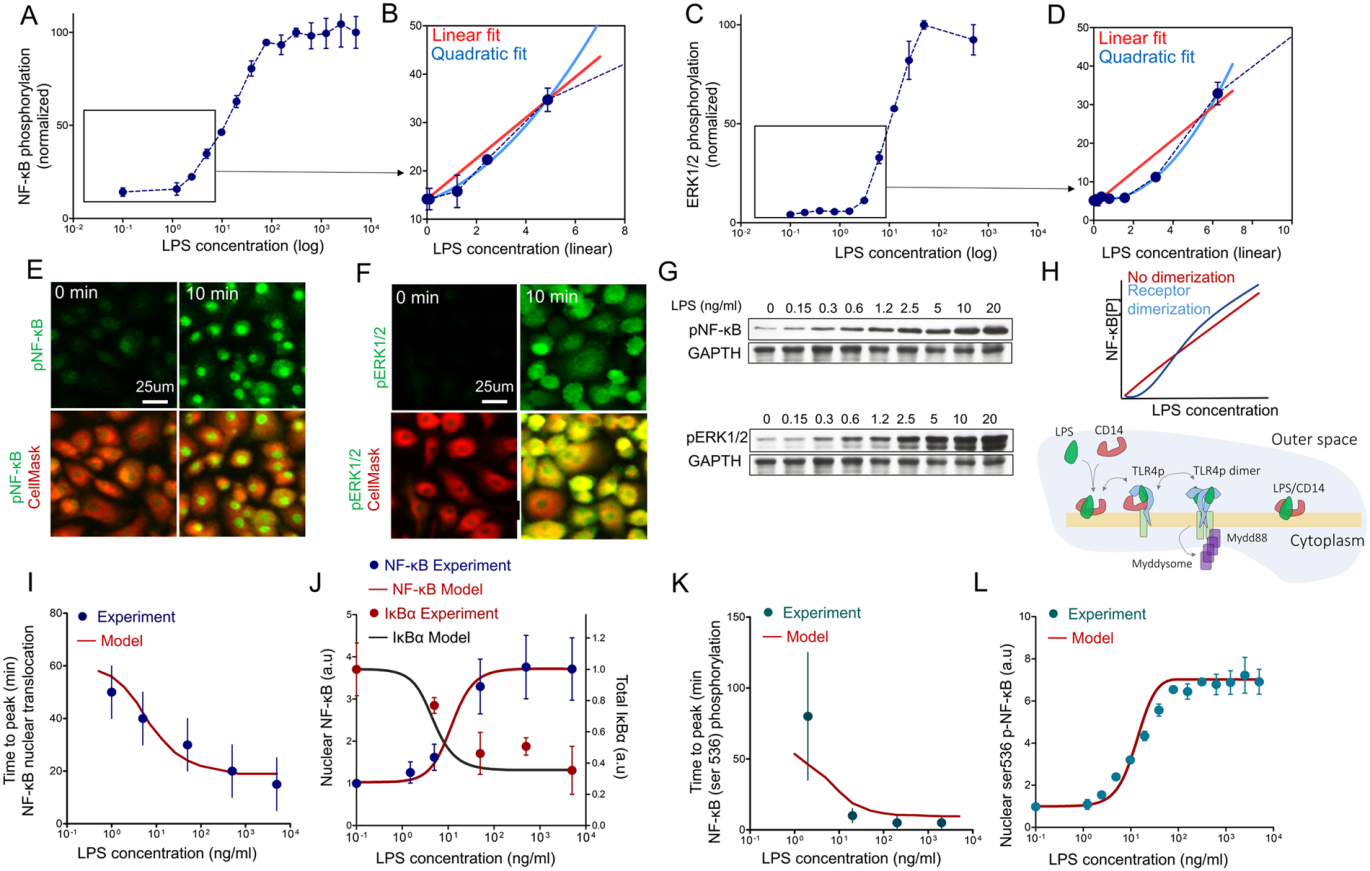
LPS activation threshold during early phosphorylation events. (**A**) Dependence of maximum intracellular amplitude of NF-κB phosphorylation (ser 536 p65) on LPS concentration as measured by flow cytometry in BMMϕ (n = 4 biological replicates). The same dependence is presented at the linear scale (**B**) at LPS-concentrations near threshold values. Red line – linear fit of phosphorylation signal. Blue line – quadratic fit of the signal. (**C**) Dependence of maximum intracellular amplitude of ERK1/2 phosphorylation (thr202/204) on LPS concentration measured by flow cytometry in BMMϕ (n = 4 biological replicates). The same dependence is presented at the linear scale (**D**) at LPS-concentrations near the threshold. (**E**,**F**) Confocal images of phosphorylated NF-κB (ser 536 p65, green) (**E**) and ERK1/2 (thr202/204, green) (**F**) intracellular distribution in bone marrow derived macrophages activated with LPS (100 ng/ml) taken before (0 min) and 10 min after activation. Red – CellMask^TM^ Deep Red staining of cell cytosol. (**G**) Western blot of phosphorylated NF-κB (ser 536 p65) and ERK1/2 (thr202/204) taken 10 min after activation with indicated concentrations of LPS and values (columns) normalized to GAPDH signal. Full-length blots are presented in Supplementary Fig. S16. (**H**) Dependence of NF-κB phosphorylation on LPS concentration as calculated in the model with the addition of TLR4 receptor dimerization processes (Blue line) or without (red line). And schematic description of TLR4 receptor dimerization process implemented in the model. (**I**) Time to peak response in the kinetics of NF-κB nuclear translocation depending on LPS concentration, model calculation (solid line) and experimental data (dots) are represented. (**J**) Amplitude of NF-κB nuclear translocation depending on LPS concentration, confocal microscopy data (dots) and model calculation (solid line). (**K**) Time to peak response in the kinetics of phosphorylated NF-κB (ser 536 p65) depending on LPS concentration. Experimental data (dots) and model calculations (solid line). (**L**) Amplitude of intracellular phosphorylated-NF-κB signal depending on LPS concentration (dots) and model calculation (solid line).

Initial signaling events in macrophages strongly depended on the concentration of the TLR4 agonist (Fig. 7). We studied the activation dependence of two main TLR4-related signaling pathways (NF-κB and ERK1/2 (MAPK) kinase pathways) on the concentration of TLR4 agonist. With LPS concentrations ranging from 0 to 5000 ng/ml, the accumulation of intracellular phosphorylated NF-κB (Fig. 7E) and ERK1/2 (Fig. 7F) increased non-linearly, demonstrating an activation threshold of 1–2 ng/ml and saturation above 100 ng/ml (Fig. 7A,C). More sensitive western blot measurements demonstrated no activation below 0.3 ng/ml of LPS (Fig. 7G). The cell’s response to a low concentration of agonist could be reliably fitted with a quadratic, rather than linear function (Fig. 7B,D). This allows us to suggest that at least a second power of cooperativity should exist in the system of signal initiation.

We added a direct description of the TLR4 dimerization process following LPS binding. This is a common and native property of many receptors and has been intensively studied for TLR4 receptor formation and activation^41–43^. Dimerization determines the ability of the receptor for self-induced transduction of the activating signal^44^. It is unclear why other model of TLR4 activation did not include that important step. This simple and intuitive addition allowed us to account for the quadratic activation threshold for LPS activation (Fig. 7H). Without receptor dimerization, the model predicts a linear dependence of NF-κB activation and IKK phosphorylation on LPS concentration (Fig. 7H), which contradicted our experimental findings (Fig. 7B,D).

After including pre-activated basal nuclear NF-κB pre-activation and TLR4 dimerization step, the model almost correctly described our experimental results in primary bone marrow-derived macrophages (Fig. 7I–L). In particular, our model correctly predicted both the shift of the time to peak position (Fig. 7I,K), the NF-κB amplitude changes and activation threshold (Fig. 7J,L) depending on LPS-concentration for kinetics of phosphorylation and nuclear translocation.

We should note that, according to our experiments, the peak time for NF-κB translocation was strongly dependent on LPS-concentration (Fig. 7I), whereas in the same range of agonist concentration the peak time for NF-κB phosphorylation insignificantly depended on LPS and immediately increases below 2 ng/ml (Fig. 7K). Both effects were correctly predicted by our model (Fig. 7I,K).

## Discussion

NF-κB signaling is a good historical example in the field of computational biology of how mathematical models can forecast and explain system behaviors – from the prediction of NF-κB oscillation, to explanations of the influence of cell-to-cell variability in digital experimental observations^5,8,15^. In this study, we accurately applied a vast body of knowledge of TLR4 activation models collected from studies on immortalized cell lines, to describe the activation of primary non-transformed macrophages with a TLR4 agonist. The determination of the model’s accuracy was translated into biological meaning – the model of TLR4/NF-κB signaling should accurately describe the dependencies of cell activation kinetics on the concentration of external signals, like LPS. On the basis of this knowledge, we made a first attempt to describe the full variety of changes in the kinetics of NF-κB translocation and NF-κB phosphorylation dependent on LPS concentration in primary macrophages. Kinetic parameters of the constructed model were imposed with the following stringent restrictions: they must simultaneously fit kinetic and concentration changes of NF-κB, IKK phosphorylation and IκBα degradation (Fig. 1).

It is well known that at LPS concentrations lower than 1 ng/ml the system responds digitally or does not respond at all, thus representing an activation threshold^45,46^. We observed a clear non-linear activation curve (phosphorylation) in both systems of NF-κB and MAPK cascades (Fig. 7). Surprisingly, such dependence was missed by previous TLR4 signaling models. We demonstrated that by adding TLR4 receptor dimerization, it is possible to account for the quadratic cooperativeness seen in the experiment (Fig. 7D). Although cooperative myddosome formation can naturally create a threshold in the MyD88 branch of the signaling even without receptor dimerization^8^, taken together with the almost linear TRIF associated path^8,13,24,47^, the mathematical description could not predict such a threshold without TLR4 dimerization. Thus, we can extend our findings and predict thresholds in other receptor systems where dimerization is a necessary event for further signal transduction, such as other TLRs^48^, NODs^49^ or GPCRs^50^.

Primary mouse BMMϕ and peritoneal macrophages showed very rapid NF-κB translocation kinetics in comparison with immortalized cell lines^6,8,14^. Here, we showed that peak NF-κB nuclear translocation is reached within 10 min after LPS priming (Fig. 1B). NF-κB phosphorylation at serine 536 peaked at 5–10 min (Fig. 1A) and was accompanied by nuclear localization of the phosphorylated NF-κB (Fig. 1B). Upon activation with LPS, almost 90% of all NF-κB is localized in the nucleus of macrophages (Fig. 4A, whereas only 60% of NF-κB was localized in the nucleus of the RAW 264.7 cell line (Fig. 4B). Mathematical modeling revealed that such fast activation could be regulated by the strength of IKK complex activation. The maximum activity and the kinetics’ shape of IKK phosphorylation determines the subsequent NF-κB activation kinetics (Fig. 2C,D), which has been generally explored since the work of^24^. Therefore, major differences in NF-κB kinetics can be associated with attenuated IKK activation in immortalized cell lines.

Survival of tissue macrophages is dependent on the constitutive activation of NF-κB^51–53^. Moreover, basal NF-κB levels can be regulated by numerous factors^54,55^. Simple stresses, as well as growth factors such as GM-CSF, can induce NF-κB activation^56–58^. In our experiments, primary macrophages had a significantly higher level of nuclear NF-κB in comparison to RAW 264.7 cells cultured under the same conditions (Fig. 4). The vast majority of primary macrophages had almost 25–30% of the total intracellular NF-κB located in the nucleus. Despite the known ability of constitutive NF-κB activity to tune macrophage activation^59^, independent intrinsic analysis of model reactions revealed that such basal NF-κB levels can significantly accelerate its’ translocation to the nucleus at low LPS concentrations: a kinetic peak can be shifted from 110 min to 50 min by varying the basal NF-κB level from 0 to 30% (Fig. 3A,B). Thus, we present a possible mathematical explanation for the phenomenon of accelerated macrophage response after low-dose LPS stimulation^60^. It is generally accepted that, for kinetic analysis, the control (baseline) levels, experimentally measured in non-activated cells, are subtracted from “response” values and treated as external noise. Here, we show that the basal activity of intracellular kinases and transcription factors does matter. Taking this into consideration, we were able to significantly increase the agreement between the model and the experimental data (Fig. 3B) and accurately describe the shift in kinetics of NF-κB translocation and phosphorylation in response to LPS concentration (Fig. 7I–L)^59^. Furthermore, we showed that RAW 264.7 cells, having a much lower basal nuclear NF-κB level, respond more slowly, thereby substantiating the model’s prediction (Fig. 5A). Mouse peritoneal exudate macrophages had highest basal NF-κB among all studied cell cultures and responded faster (Fig. 5C). We intentionally did not focus our attention on the processes that could generate the constitutive basal NF-κB level. Instead, we chose to manually add constitutive phosphorylation of the IKKα/β complex from an unknown source^61,62^. According to the model equations, NF-κB cannot be found in a free state without IKK activation, which triggers degradation of IκB and translocation of NF-κB into the nucleus (Fig. 3C). Very small level of pre-activated IKK (less than 1%) and decreased amount of IκB inhibitor lets the system to faster react on the external signal (Fig. 3C).

Although IKK could be triggered by constitutive activation of upstream kinases, such as IRAKs or TRAF6, this does not change the consequent calculations and conclusions. We speculate that increased basal NF-κB activity could be characteristic of primary macrophages, as within the tissue milieu they are subject to the influence of dozens of growth factors and cytokines. Mouse peritoneal exudate macrophages had highest basal NF-κB, among all studied cell cultures. We suggest that basal activity of the various pathways could provide an important regulation in different signaling pathways. Such correlations were observed in other cell types and biological processes^63,64^.

NF-κB phosphorylation can selectively regulate NF-κB transcriptional activity by modulating DNA binding in a sequence specific manner, providing regulatory mechanism for expression of different cytokines^19^. The phosphorylation of p65 induces a conformational change, which impacts p65 ubiquitination and stability, as well as protein protein interactions^65^. In macrophages, the phosphorylation of p65 at S536 by IKKα increases p65 turnover, thereby reducing NF-κB activity and supporting the resolution of inflammation. Phosphorylated p65 has a lower affinity for IκBα, which results in increased nuclear translocation and accumulation of p65^66^.

To our knowledge, we present the first model that accounts for the NF-κB phosphorylation process in NF-κB nuclear translocation. This could be a starting point to further extend NF-κB signaling models considering changes in phosphorylation-dependent protein interactions^67^ and gene induction. Guided by experimental measurements of intracellular phosphorylation, we found that the kinetics of p65 phosphorylation representing rapid activation and attenuation cannot be described by the simple addition of phosphorylating/dephosphorylating processes. This is due to the high levels of active IKK, at least during the 2 hours of our observation. Thus, our model predicts the importance of accounting negative regulation of NF-κB phosphorylation.

There is a vast range of known negative regulators within the NF-κB signaling module^35^. Most of these are induced transcriptionally and disrupt signal transduction from the receptor to the nucleus. They can directly bind active NF-κB subunits like IκBα^9^, trigger IRAK dephosphorylation through IRAK-M^68^, induce the deubiquitination of signaling proteins (A20,^69^), regulate nuclear activity (CITED2,^70^) or participate in the degradation of signaling proteins, such as TRIM30a, PDLIM2, SOCS-1, COMMD1 and others^35,71^. The unifying property of all of these regulators is that they completely deactivate the NF-κB system. That is why none of them can explain the more specific attenuation of NF-κB phosphorylation without complete disruption of NF-κB nuclear translocation to the nucleus. According to our experimental data, NF-κB persists in the nucleus long after the inhibition of NF-κB phosphorylation at serine 536 (Fig. 1). Our model predicts the presence of more delicate regulation of NF-κB phosphorylation, possibly through proteins that directly associate with NF-κB dephosphorylation, like WIP1^40^. Accounts of such interactions are crucially important for understanding the NF-κB activation processes outside of its’ nuclear translocation events.

## Methods

### Ethics Statement

The study was carried out in accordance with the Russian Guidelines for the Care and Use of Laboratory Animals. The protocol was approved by the local ethics committee of the Institute of Immunology of FMBA Russia (Order of November 12, 2015), certified by the Local Ethics Committee (Resolution 4/17 of July 13, 2017).

### Mice

Female eight- to ten-week-old BALB/c or C57BL mice were obtained from the breeder Stolbovaya (Moscow, Russia) and fed standard rodent food under standard animal house conditions in the vivarium of the National Research Center Institute of Immunology FMBA, Moscow, Russia.

### Reagents

Lipopolysaccharide, TLR4 tested (B5:055, Sigma), antibodies against RelA/p65 (Cell Signaling, D14E12), antibodies against RelA/p65 (ab16502 Abcam), phosphorylated RelA/p65 at ser 536 (Cell Signalling, 93H1), IκBα (Cell Signalling, 112B2), phosphorylated ERK1 and 2 (thr 202/204, Cell Signalling, #9101), phosphorylated IKKα/β (ser176/180, Cell Signaling, 16A6), Goat anti-Rabbit AF488 (Invitrogen), CD115 conjugated with FITC (BD), F4/80 conjugated with APC (BD), CD11b conjugated with BV510, I-A conjugated with FITC, CellMask™ Deep Red Plasma membrane Stain (ThermoFisher), ProLong® Gold Antifade Mountant with DAPI (ThermoFisher).

### Cell cultures

All cell cultures were incubated in a complete medium (CM) based on DMEM with 25 mM HEPES supplemented with a cocktail of nonessential amino acids, 10% fetal bovine serum (FBS, HyClone, Cat # SV30160.03 endotoxin level ≤10 EU/ml), 2 mM L- glutamine, 1 mM sodium pyruvate, and 10 μg/ml gentamycin at 37оС in a 5% СO2 humidified atmosphere (all culture supplements were obtained from PanEco, Russia).

Bone marrow derived-macrophages were obtained *in vitro* by culturing bone marrow cells of BALB/c mice with a granulocyte-macrophage colony-stimulating factor (GM-CSF)^20^. Bone marrow was washed out from the femurs and the tibias, erythrocytes removed by osmotic shock, nuclear cells washed twice in PBS (Amresco, E404), followed by cultivation in a complete medium supplemented with 10 ng/ml GM-CSF (Sigma) for 7 days as described, media changed at day 4. After 7 days of culture, the adherent cells (Macrophages) were detached by incubation in a Versene solution (EDTA, PanEco) for 30 min at 37 °С. Cells washed with PBS supplemented with 1% glucose, 10 mM HEPES, and 0.5% BSA, then re-suspended in CM. Viability and purity were assayed with flow cytometry. Viable cell (>80% by DAPI staining) comprised 95–98% of macrophages revealed by staining with CD11b, I-A, CD115.

Peritoneal macrophages were obtained by washing of the peritoneal cavity of BALB/c mice with PBS. The cells were pelleted by centrifugation, resuspended in CM, and cultured for 18–20 h at 37oC in a humidified atmosphere of 5% СO2. Then non-adhesive cells were gently washed away with warm PBS. The remaining adherent cells comprised over 90% of macrophages assayed by flow cytometry analysis with CD115 staining.

RAW 264.7 murine macrophage cell line were a gift from Dr. Alexander Sapozhnikov (Shemyakin and Ovchinnikov Institute of Bioorganic Chemistry, Moscow, Russia) and cultured at the same conditions as bone marrow derived macrophages.

### Confocal imaging

Cells were plated on eight-well glass bottom µ-slides (SPL life science) at the density of 5 × 10^4^ per well and incubated in a complete DMEM overnight. Next day the appropriate concentration of LPS were added to the each well. At the each time point all media were removed from the well and 4% Paraformaldehyde were added immediately. This step was made quickly to minimize time error, which was calculated to be ± 20 sec. Cells fixed for 20 min, then washed with PBS and permeabilized in cold pure ethanol at −20C for 30 min, washed several times with PBS and stained with antibodies. Prior to imaging cells stained with Cell Mask Deep red plasma membrane stain (Invitrogen) and covered with Prolong antifade solution with DAPI (Invitrogen). Cells were imaged by Axio Observer Z1 microscope (Zeiss) with a QuantEM 512SC camera (Photometrics) and 405, 488, 635 nm lasers with 20x or 100x oil immersion objectives.

NF-κB translocation were measured as described in^21^, with slight modifications. Briefly, fluorescent images of cells were taken at the focal layer of the nucleus (positioned with DAPI), the mean fluorescent signal of NF-κB located in nucleus area (located with DAPI fluorescence) was calculated for each individual cell using ImageJ software.

### Flow cytometry

Intracellular accumulation of phosphorylated NF-κB, phosphorylated IKKα/β, phosphorylated ERK1/2 and degradation of IκBα were measured by flow cytometry on FACS Aria II (BD Biosciences) as described in^72^ with little modifications. Cells were harvested at the day of activation and resuspended in complete medium in Eppendorf tubes – 50000 per tube. Two hours later, an appropriate concentration of LPS were added to each tube. At every time point, cells were immediately resuspended in 4% paraformaldehyde for 30 min. Then cells were washed and permeabilized in cold pure ethanol at −20 for 30 min, washed several times with PBS. Fixed cells were stained with primary antibodies for 2 hours, then washed and stained with secondary AF488 conjugated antibodies for 1 hour.

### Western blot

BMMϕ were solubilized in Cell Extraction Buffer, (Invitrogen) supplemented with protease/phosphatase inhibitor cocktail (Sigma). Cell debris was pelleted at 14,000 rpm for 10 min at 40 C. The protein concentration in lysate was measured using Pierce BCA Protein Assay Kit (Thermo Fisher Scientific). Lysates were mixed with SDS sample buffer containing reducing agent, and proteins were heat denatured at 90 °C for 5 minutes. From 10 µg of the total proteins per lane was used to sample load on home-made 8% SDS-PAGE gel. Proteins were resolved in Tris-Glycine/SDS running buffer and then transferred to Amersham™ Hybond® P Western blotting membrane (Amersham). Blots were incubated with primary rabbit IgG antibodies against either phosphorylated pErk1/2 (T202/204) (Cell Signalling), phosphorylated RelA/p65 at ser 536 (Cell Signalling) GAPDH (Abcam) under constant agitation. Membranes were probed with the secondary goat anti-rabbit IgG antibody conjugated to horse radish peroxidase (Sigma). Bands were visualized by exposing blotting membrane on Amersham X-ray films (Amersham, USA) Comparative densitometry of the bands was carried out with ImageJ software and normalized to the density of the respective GAPDH.

### qRT PCR analysis

The level of mRNA expression for *TNFα* (F: CATCTTCTCAAAATTCGAGTGACAA, R: TGGGAGTAGACAAGGTACAACCC, FAM-CACGTCGTAGCAAACCACCAAGTGGA-RTQ) were evaluated using quantitative RT-PCR. Cells were seeded on 96-well plate (30000 cells per well), as described in cell culture section, and treated with LPS for an appropriate time exposure. Then cells were rinsed with PBS and total RNA was extracted single-step method of RNA isolation by acid guanidinium thiocyanate–phenol–chloroform extraction. Total RNA, obtained using RNA-extraction kit (Syntol, Russia) were further treated with DNAse (Thermo Scientific, USA). cDNA was obtained with cDNA Synthesis Kit according to manufacturer’s instructions (Syntol, Russia). At each cycle, the fluorescence of FAM was measured to monitor progression. Expression of target RNA was normalized to expression of β-actin (F: AGAGGGAAATCGTGCGTGAC, R: CAATAGTGATGACCTGGCCGT, FAM-CACTGCCGCATCCTCTTCCTCCC-RTQ).

### Mathematical model

The ODE model was constructed to describe TLR4 induced NF-κB activation and phosphorylation in bone-marrow derived macrophages (S1 Methods). It includes processes of (i) ligand recognition (LPS), (ii) formation of dimer receptor complex, (iii) signal transduction through MyD88 and TRIF adaptors via TRAF6/TAK1, (iv) activation of IKK kinase, which in turn enables (v) the phosphorylation and nucleus translocation of NF-κB transcription factor and synthesis induction of IκB and TNFα genes. The final model consisted of 53 Species and contains 95 reactions (S4Table).

All equations were solved numerically using Copasi package (//copasi.org) or Wolfram Mathematica 10 (Wolfram Research, Inc.) in the form of differential equations. Experimental data fitting was performed on median values using Ordinary Least Squares error function with weights as a data variance with Nelder-Mead or Evolution programming algorithms (Copasi).

Model was deposited in BioModels (http://www.ebi.ac.uk) and assigned the identifier MODEL1809230001

### Statistical analysis

The data are displayed as median ± SD or ±25%/75% quantiles (if data is distributed) of values distribution within cells processed from high number of confocal images. Goodness of model fit were calculated using Chi-square test. p < 0.05 was used to establish statistical significance. Gauss nonlinear fit of experimental data, Cramer-Von Mises criteria and Kolmogorov-Smirnov test were performed by Wolfram Mathematica packages.

## Data Availability

All data analysed during this study are included in this published article (and its Supplementary Information files). Model was deposited in BioModels (http://www.ebi.ac.uk) and assigned the identifier MODEL1706250000.

## Electronic supplementary material

Supplementary info and model description

## References

[1] Kawai T, Akira S (2010). The role of pattern-recognition receptors in innate immunity: update on Toll-like receptors. Nat. Immunol..

[2] Sharif O, Bolshakov VN, Raines S, Newham P, Perkins ND (2007). Transcriptional profiling of the LPS induced NF-kB response in macrophages. BMC Immunology.

[3] Nilsson R (2006). Transcriptional network dynamics in macrophage activation. Genomics.

[4] Duque, G. A. & Descoteaux, A. Macrophage cytokines: Involvement in immunity and infectious diseases. *Frontiers in Immunology* **5** (2014). 10.3389/fimmu.2014.00491 PMC4188125 25339958

[5] Hoffmann, A., Levchenko, A., Scott, M. L. & Baltimore, D. The IkB - NFkB Signaling Module: Temporal Control and Selective Gene Activation. *Science (80-*.*)*. **1241** (2002). 10.1126/science.1071914 12424381

[6] Covert MW, Leung TH, Gaston JE, Baltimore D (2005). Achieving stability of lipopolysaccharide-induced NF-kappaB activation. Science.

[7] Cheong R, Hoffmann A, Levchenko A (2008). Understanding NF-kappaB signaling via mathematical modeling. Mol. Syst. Biol..

[8] Cheng Z, Taylor B, Ourthiague DR, Hoffmann A (2015). Distinct single-cell signaling characteristics are conferred by the MyD88 and TRIF pathways during TLR4 activation. Sci. Signal..

[9] Fagerlund, R. *et al*. Anatomy of a negative feedback loop: The case of IκB. *J*. *R*. *Soc*. *Interface* **12** (2015). 10.1098/rsif.2015.0262 PMC4614452 26311312

[10] Oda K, Kitano H (2006). A comprehensive map of the toll-like receptor signaling network. Mol. Syst. Biol..

[11] Kawai T, Akira S (2007). TLR signaling. Semin Immunol.

[12] Moreno R, Sobotzik JM, Schultz C, Schmitz ML (2010). Specification of the NF-kB transcriptional response by p65 phosphorylation and TNF-induced nuclear translocation of IKKe. Nucleic Acids Res.

[13] Sharp, G. C., Ma, H., Saunders, P. T. K. & Norman, J. E. A Computational Model of Lipopolysaccharide-Induced Nuclear Factor Kappa B Activation: A Key Signalling Pathway in Infection-Induced Preterm Labour. *PLoS One* **8** (2013). 10.1371/journal.pone.0070180 PMC3736540 23936158

[14] Gutschow MV (2013). Single-Cell and Population NF-κB Dynamic Responses Depend on Lipopolysaccharide Preparation. PLoS One.

[15] Nelson DE (2004). Oscillations in NF-κB signaling control the dynamics of gene expression. Science (80-.)..

[16] Caldwell AB, Birnbaum HA, Cheng Z, Hoffmann A, Vargas JD (2014). Network dynamics determine the autocrine and paracrine signaling functions of TNF. Genes Dev..

[17] Lee TK (2009). A Noisy Paracrine Signal Determines the Cellular NF-kB Response to Lipopolysaccharide. Sci. Signal..

[18] Padwal MK, Sarma U, Saha B (2014). Comprehensive logic based analyses of toll-like receptor 4 signal transduction pathway. PLoS One.

[19] Christian F, Smith E, Carmody R (2016). The Regulation of NF-κB Subunits by Phosphorylation. Cells.

[20] Helft J (2015). GM-CSF Mouse Bone Marrow Cultures Comprise a Heterogeneous Population of CD11c+ MHCII+ Macrophages and Dendritic Cells. Immunity.

[21] Trask OJ (1988). Nuclear Factor Kappa B (NF-κB) Translocation Assay Development and Validation for High Content Screening. Assay Guid. Man..

[22] Werner SL (2008). Encoding NF-κB temporal control in response to TNF: distinct roles for the negative regulators IκBα and A20. Genes Dev..

[23] Selvarajoo K (2006). Discovering differential activation machinery of the Toll-like receptor 4 signaling pathways in MyD88 knockouts. FEBS Lett..

[24] Werner SL (2005). Stimulus Specificity of Gene Expression Programs Determined by Temporal Control of IKK Activity. Science (80-.)..

[25] Phelps CB, Sengchanthalangsy LL, Malek S, Ghosh G (2000). Mechanism of κB DNA binding by Rel/NF-κb dimers. J. Biol. Chem..

[26] Bergqvist S (2009). Kinetic enhancement of NF-kappaBxDNA dissociation by IkappaBalpha. Proc. Natl. Acad. Sci. USA.

[27] Bosisio D (2006). A hyper-dynamic equilibrium between promoter-bound and nucleoplasmic dimers controls NF-kappaB-dependent gene activity. EMBO J..

[28] Sakai J (2017). Lipopolysaccharide-induced NF-κB nuclear translocation is primarily dependent on MyD88, but TNFα expression requires TRIF and MyD88. Sci. Rep..

[29] Gottschalk RA (2016). Distinct NF-κB and MAPK Activation Thresholds Uncouple Steady-State Microbe Sensing from Anti-pathogen Inflammatory Responses. Cell Syst..

[30] Takeda K, Akira S (2005). Toll-like receptors in innate immunity. Int. Immunol..

[31] Wan F, Lenardo MJ (2009). Specification of DNA Binding Activity of NF- B Proteins. Cold Spring Harb. Perspect. Biol..

[32] Hochrainer K, Racchumi G, Anrather J (2013). Site-specific phosphorylation of the p65 protein subunit mediates selective gene expression by differential NF-kB and RNA polymerase II promoter recruitment. J. Biol. Chem..

[33] Sasaki CY, Barberi TJ, Ghosh P, Longo DL (2005). Phosphorylation of Re1A/p65 on serine 536 defines an IkBa- independent NF-kB pathway. J. Biol. Chem..

[34] Viatour P, Merville MP, Bours V, Chariot A (2005). Phosphorylation of NF-κB and IκB proteins: Implications in cancer and inflammation. Trends Biochem. Sci..

[35] Renner F, Schmitz ML (2009). Autoregulatory feedback loops terminating the NF-kappaB response. Trends Biochem. Sci..

[36] Enesa K, Evans P (2014). The biology of A20-like molecules. Adv. Exp. Med. Biol..

[37] Zwergal A (2006). C/EBP-beta Blocks p65 Phosphorylation and Thereby NF-kappa-B-Mediated Transcription in TNF-tolerant Cells. J Immunol.

[38] Yang J, Fan GH, Wadzinski BE, Sakurai H, Richmond A (2001). Protein Phosphatase 2A Interacts with and Directly Dephosphorylates RelA. J. Biol. Chem..

[39] Li H-Y (2008). Deactivation of the kinase IKK by CUEDC2 through recruitment of the phosphatase PP1. Nat. Immunol..

[40] Chew J (2009). WIP1 phosphatase is a negative regulator of NF-kappaB signalling. Nat. Cell Biol..

[41] Park BS (2009). The structural basis of lipopolysaccharide recognition by the TLR4-MD-2 complex. Nature.

[42] Tsukamoto H, Fukudome K, Takao S, Tsuneyoshi N, Kimoto M (2010). Lipopolysaccharide-binding protein-mediated Toll-like receptor 4 dimerization enables rapid signal transduction against lipopolysaccharide stimulation on membrane-associated CD14-expressing cells. Int. Immunol..

[43] Bovijn C (2012). Identification of interaction sites for dimerization and adapter recruitment in toll/interleukin-1 receptor (TIR) domain of toll-like receptor 4. J. Biol. Chem..

[44] Park BS, Lee J-O (2013). Recognition of lipopolysaccharide pattern by TLR4 complexes. Exp. Mol. Med..

[45] Velickovic CT (2008). Low levels of endotoxin enhance allergen-stimulated proliferation and reduce the threshold for activation in human peripheral blood cells. Int. Arch. Allergy Immunol..

[46] Kellogg, R. A., Tian, C., Lipniacki, T., Quake, S. R. & Tay, S. Digital signaling decouples activation probability and population heterogeneity. *Elife* **4** (2015). 10.7554/eLife.08931 PMC4608393 26488364

[47] Rivière, B., Epshteyn, Y. & Swigon, D. V. Y. A simple mathematical model of signaling resulting from the binding of lipopolysaccharide with Toll-like receptor 4 demonstrates inherent preconditioning behavior. **217**, 19–26 (2010). 10.1016/j.mbs.2008.10.002 PMC2651675 18950647

[48] Yamamoto M, Takeda K (2010). Current views of toll-like receptor signaling pathways. Gastroenterol. Res. Pract..

[49] Caruso R, Warner N, Inohara N, Nunez G (2014). NOD1 and NOD2: Signaling, host defense, and inflammatory disease. Immunity.

[50] Bai M (2004). Dimerization of G-protein-coupled receptors: Roles in signal transduction. Cellular Signalling.

[51] Pagliari LJ, Perlman H, Liu H, Pope RM (2000). Macrophages require constitutive NF-kappaB activation to maintain A1 expression and mitochondrial homeostasis. Mol. Cell. Biol..

[52] Ellrichmann G (2012). Constitutive activity of NF-kappa B in myeloid cells drives pathogenicity of monocytes and macrophages during autoimmune neuroinflammation. J. Neuroinflammation.

[53] Bhakar aL (2002). Constitutive nuclear factor-kappa B activity is required for central neuron survival. J Neurosci.

[54] Hupalowska a, Pyrzynska B, Miaczynska M (2012). APPL1 regulates basal NF- B activity by stabilizing NIK. J. Cell Sci..

[55] Li Q (2005). Enhanced NF-kappaB activation and cellular function in macrophages lacking IkappaB kinase 1 (IKK1). Proc. Natl. Acad. Sci. USA.

[56] Tam, A. B., Mercado, E. L., Hoffmann, A. & Niwa, M. ER Stress Activates NF-κB by Integrating Functions of Basal IKK Activity, IRE1 and PERK. *PLoS One* **7** (2012). 10.1371/journal.pone.0045078 PMC3482226 23110043

[57] Meads MB, Li Z-W, Dalton WS (2010). A novel TNF receptor-associated factor 6 binding domain mediates NF-kappa B signaling by the common cytokine receptor beta subunit. J. Immunol..

[58] Yu M, Qi X, Moreno JL, Farber DL, Keegan AD (2011). NF-κB signaling participates in both RANKL- and IL-4-induced macrophage fusion: receptor cross-talk leads to alterations in NF-κB pathways. J. Immunol..

[59] Zambrano, S., Bianchi, M. E. & Agresti, A. High-throughput analysis of NF-κB dynamics in single cells reveals basal nuclear localization of NF-κB and spontaneous activation of oscillations. *PLoS One* **9** (2014). 10.1371/journal.pone.0090104 PMC3942427 24595030

[60] Saperstein S, Chen L, Oakes D, Pryhuber G, Finkelstein J (2009). IL-1beta augments TNF-alpha-mediated inflammatory responses from lung epithelial cells. J. Interferon Cytokine Res..

[61] Gasparian AV (2002). The role of IKK in constitutive activation of NF-κB transcription factor in prostate carcinoma cells. J. Cell Sci..

[62] Israël A (2010). The IKK Complex, a Central Regulator of NF-κB Activation. Cold Spring Harb. Perspect. Biol..

[63] Macia, J. *et al*. Dynamic signaling in the Hog1 MAPK pathway relies on high basal signal transduction. *Sci. Signal*, 10.1126/scisignal.2000056 (2009). 10.1126/scisignal.2000056 19318625

[64] Di Angelantonio, S. *et al*. Basal adenosine modulates the functional properties of AMPA receptors in mouse hippocampal neurons through the activation of A1R A2AR and A3R. *Front. Cell. Neurosci*, 10.3389/fncel.2015.00409 (2015). 10.3389/fncel.2015.00409 PMC4601258 26528137

[65] Milanovic, M., Kracht, M. & Schmitz, M. L. The cytokine-induced conformational switch of nuclear factor κB p65 is mediated by p65 phosphorylation. *Biochem*. *J*, 10.1042/BJ20130780 (2014). 10.1042/BJ20130780 24175631

[66] Pringle, L. M. *et al*. Atypical mechanism of NF-κB activation by TRE17/ubiquitin-specific protease 6 (USP6) oncogene and its requirement in tumorigenesis. *Oncogene*, 10.1038/onc.2011.520 (2012). 10.1038/onc.2011.520 PMC3297677 22081069

[67] Ryo A (2003). Regulation of NF-κB Signaling by Pin1-Dependent Prolyl Isomerization and Ubiquitin-Mediated Proteolysis of p65/RelA. Mol. Cell.

[68] Du J (2014). The structure function of the death domain of human IRAK-M. Cell Commun. Signal..

[69] Pujari R, Hunte R, Khan WN, Shembade N (2013). A20-mediated negative regulation of canonical NF-κB signaling pathway. Immunol. Res..

[70] Lou X (2011). Negative feedback regulation of NF-κB action by CITED2 in the nucleus. J. Immunol..

[71] Shi M (2008). TRIM30 alpha negatively regulates TLR-mediated NF-kappa B activation by targeting TAB2 and TAB3 for degradation. Nat. Immunol..

[72] Perez OD, Krutzik PO, Nolan GP (2004). Flow cytometric analysis of kinase signaling cascades. Methods Mol Biol.

[73] Werner SL (2008). Encoding NF-kB temporal control in response to TNF: distinct roles for the negative regulators IkBa and A20. Genes Dev..

