# Supplementary material for: Elevated pre-activation basal level of nuclear NF-κB in native macrophages accelerates LPS-induced translocation of cytosolic NF-κB into the cell nucleus
Source: Sci Rep. 2019 Mar 14;9:4563. doi: 10.1038/s41598-018-36052-5 (PMC6418260; doi:10.1038/s41598-018-36052-5)
Supplement: Supplementary file 1 — Supplementary info and model description [file 41598_2018_36052_MOESM1_ESM.docx]

**Supporting information**

Elevated pre-activation basal level of nuclear NF-κB in native macrophages accelerates LPS-induced translocation of cytosolic NF-κB into the cell nucleus

**Alexander V. Bagaev^1,4*^, Anastasiya Y. Garaeva^1^, Ekaterina S. Lebedeva^1^, Alexey V. Pichugin^1^, Ravshan I. Ataullakhanov^1,5¶^, Fazly I. Ataullakhanov^2,3,4,6¶*^**

^1^National Research Center – Institute of Immunology Federal Medical-Biological Agency of Russia, Moscow, Russia

^2^Center for Theoretical Problems of Physicochemical Pharmacology, Russian Academy of Sciences, Moscow, Russia.

^3^National Scientific and Practical Center of Pediatric Hematology, Oncology and Immunology, Moscow, Russia.

^4^Department of Physics, Moscow State University, Moscow, Russia.

^5^Department of Biology, Moscow State University, Moscow, Russia

^6^Moscow Institute of Science and Technology, Dolgoprudny, Russia.

^¶^These authors contributed equally to this work

**
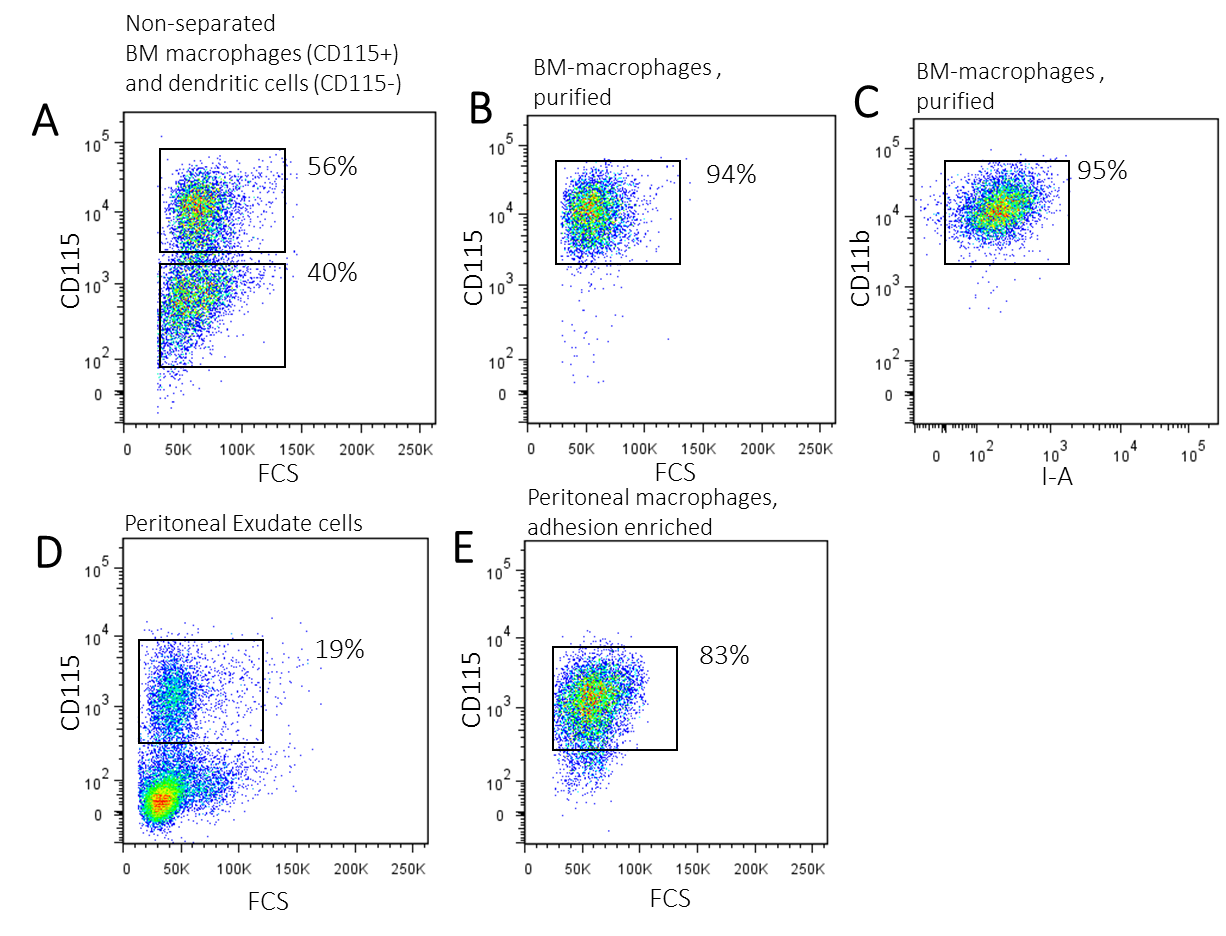
**

**Fig S1. The purity of macrophages used in the experiments, examined by flow cytometry.**

**A –** GM-CSF generated bone marrow cells contain both dendritic and macrophage cell populations if dendritic cells are not removed ^1^. Cells were stained with CD115/CD11b/I-A antibodies cocktail. Dendritic cells are loosely adhesive and can be removed from adhesive macrophages by thorough washing with PBS.

**B, C** – Purity of bone marrow derived macrophages after washing of dendritic cells with PBS. Cells were stained with CD115/CD11b/I-A antibodies cocktail.

**D –** Cells isolated from mouse peritoneal cavity were stained with CD115 antibody before macrophages enrichment.

**E** – Purity of peritoneal macrophages after their enrichment. Macrophages were enriched by 24-hours adhesion to the culture plastic and subsequent washes of non-adhesive cell types.


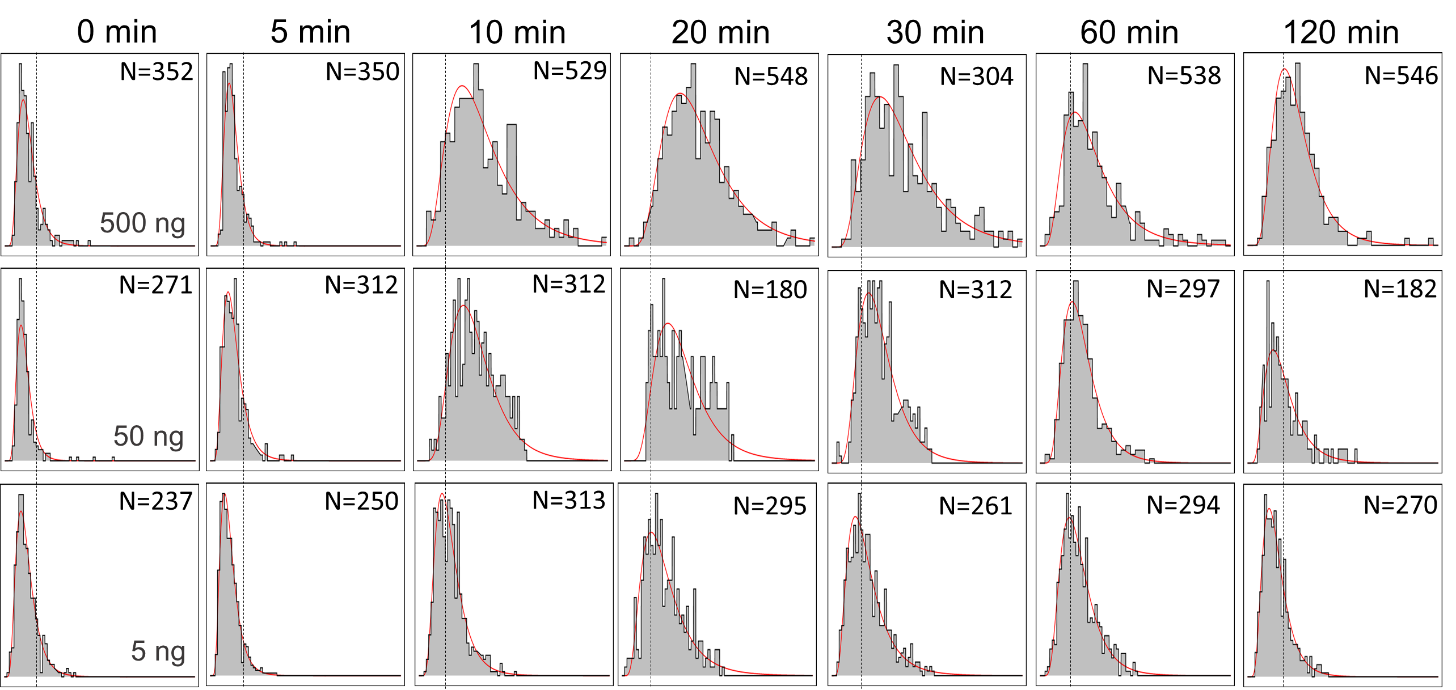


**Fig S2. NF-kB distribution within nuclei of activated macrophages fall into uni-modal log-normal distribution.** The distribution of NF-κB signal within bone marrow-derived macrophages activated with 500, 50 and 5 ng/ml of LPS, fixed at appropriate time points and stained as described in methods. Fluorescent images of cells were taken at the focal layer of the nucleus (positioned with DAPI), the mean fluorescent signal of NF-κB located in nucleus area (located with DAPI fluorescence) was calculated for each individual cell using ImageJ software. Red line – Log-normal distribution fit. (Cramer-Von Mises criteria p-value for log-normal distribution p= 0.67, for normal distribution p=0.0051).

**
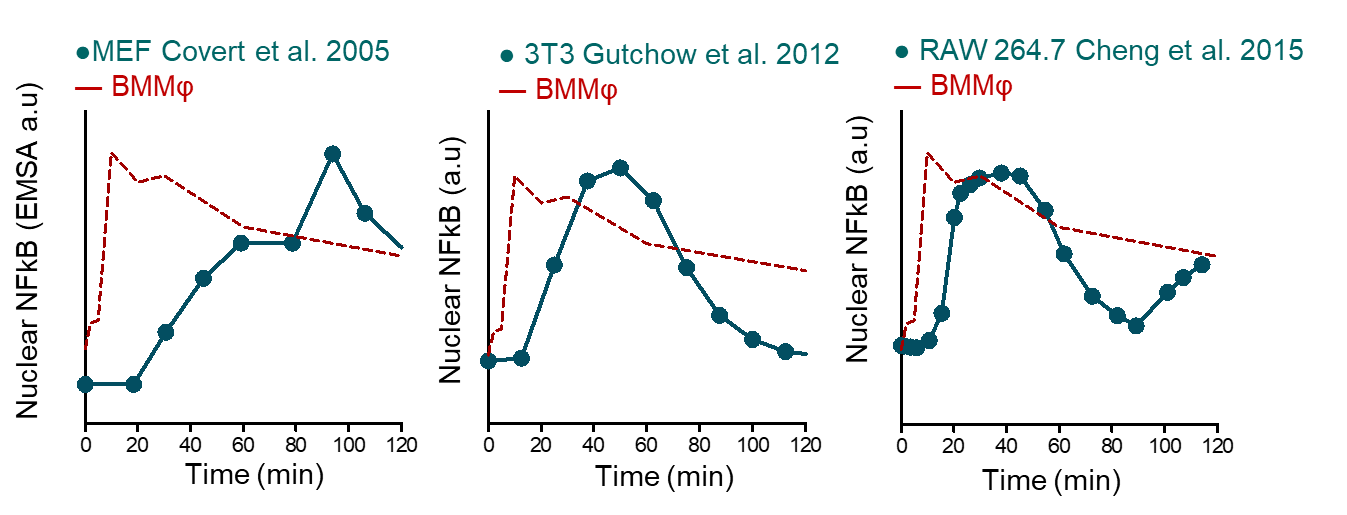
**

**Fig S3.** Comparison of NF-κB translocation kinetics (upon 500 ng/ml LPS stimulation) of primary BMMϕ (red dashed line) in our experiments with immortalized cell types (MEF, 3T3, RAW 264.7 cell lines) widely used for validation of mathematical models of TLR4-induced NF-κB translocation ^6,8,14^

**
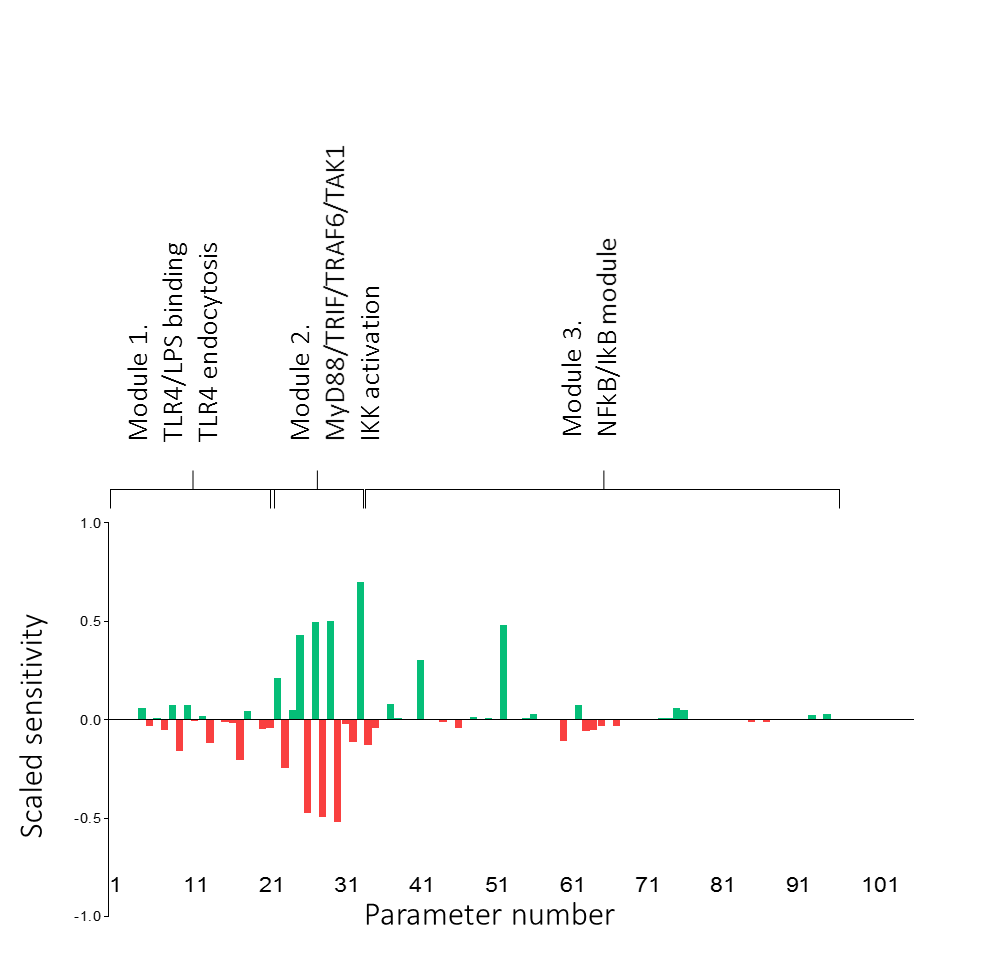
**

**Fig S4.** Scaled sensitivity score (related to the rate of NF-κB translocation) for all kinetic parameters of the model. Red values represent negative derivatives and green ones have positive derivatives.


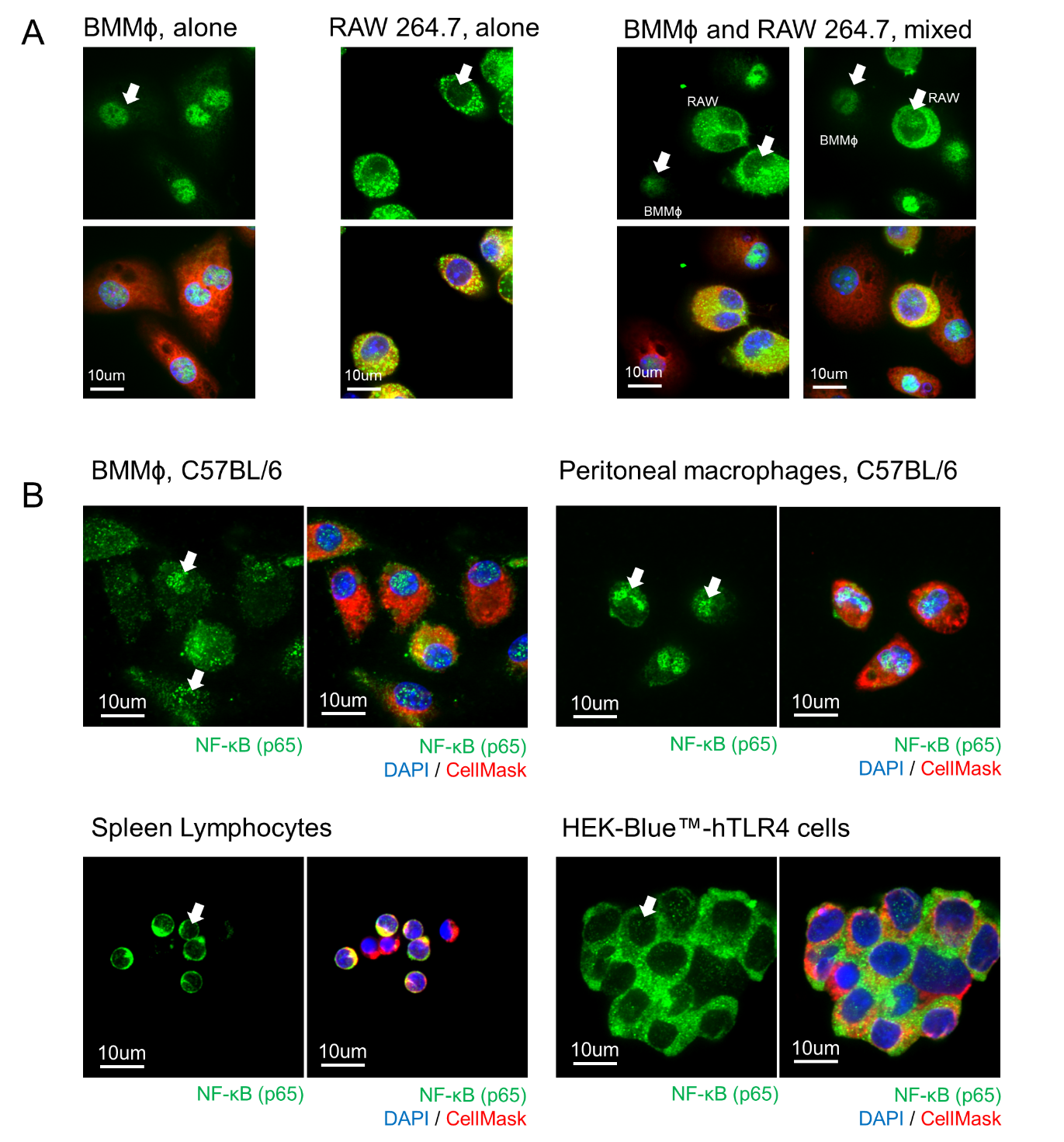


**Fig S5. Basal NF-κB staining in different cell cultures.**

1. Confocal images of NF-κB location before activation stained in macrophages isolated from mouse peritoneal cavity (BALB/c) or RAW264.7 cells cultured alone or cultured together as a mixed suspension to avoid any other possible differences in the culture method.
2. Confocal images of NF-κB location before activation with LPS in the Bone-marrow derived macrophages (BMMϕ), macrophages isolated from mouse peritoneal cavity (PEC) of C57BL/6 mice, Lymphocytes isolated from mouse spleen cells and HEK293 cell line.


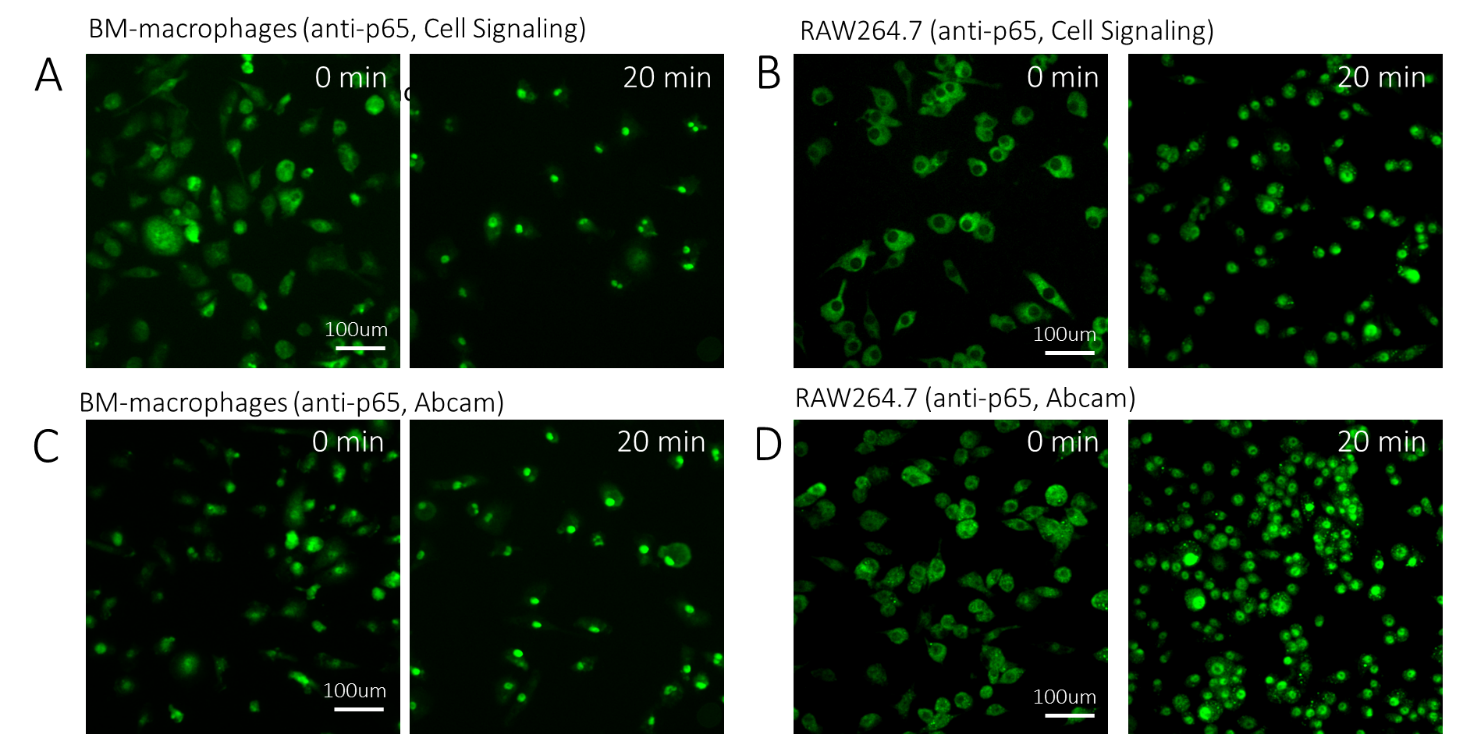


**Fig S6. NF-κB translocation analysis using two different monoclonal antibodies.**

Primary macrophages had higher basal nuclear NF-κB signal while transformed RAW264.7 macrophages had significantly lower nuclear NF-κB, independently from antibody. **A, C** - Bone marrow macrophages before and 20 min after activation with LPS (500 ng/ml) stained with rabbit monoclonal antibodies recognizing p65 subunit of NF-κB (A, Cell Signaling) or (C, Abcam). **B, D** – RAW264.7 macrophages before and 20 min after LPS (500 ng/ml) activation stained with rabbit monoclonal antibodies recognizing p65 subunit of NF-κB (B, Cell Signaling) or (D, Abcam).

**S1 Methods**

1. **Computational ODE model describing LPS induced NF-κB activation**
   1. **Model description.**

The model was constructed to describe TLR4 induced NF-κB activation in native bone marrow derived macrophages. It included processes of ligand (lipopolysaccharide) recognition, formation of dimer receptor complex and further signal transduction through TRAF6/TAK1 complex that leads to the activation of IKKα/β kinase, which in turn enables the NF-κB transcription factor phosphorylation and translocation in the cell nucleus, and induction of IkB and WIP1 (as an example of induced protein that promotes NF-κB dephosphorylation ^2^) gene transcription. Models were based on the current knowledge of TLR signaling framework ^3^, protein interactions within the TLR4 pathway ^4–6^, and up-to-date mathematical models describing Toll receptor activation (discussed below).

The major important additions were made to TLR4 signaling description:

1. Receptor dimerization process
2. Existence of a basal nuclear NF-κB level (translocation)
3. NF-κB phosphorylation by IKK complex

The final ODE model consisted of 53 Species and contains 95 reactions (S4 Table). Final model was deposited in BioModels (http://www.ebi.ac.uk) and assigned the identifier MODEL1706250000

For system simplification, we divided TLR4 pathway into 3 modules. Module 1 or ‘TLR4 module’ describes the interaction of the TLR4 receptor with LPS, receptor dimerization and endocytosis of the receptor-ligand complex. Module 2 or ‘IKK module’ connected with signal transduction in form of kinase activity to the endpoint of IKK complex (NEMO/IKKα/β) phosphorylation. Module 3 or ‘NF-κB module’ is a block of reactions describing IKK dependent catalysis of NF-κB inhibitor – IκBα (IkBβ, IkBε) - and the negative feedback loop connected with induction of IkB synthesis and NF-κB phosphorylation process (Fig S7).

**
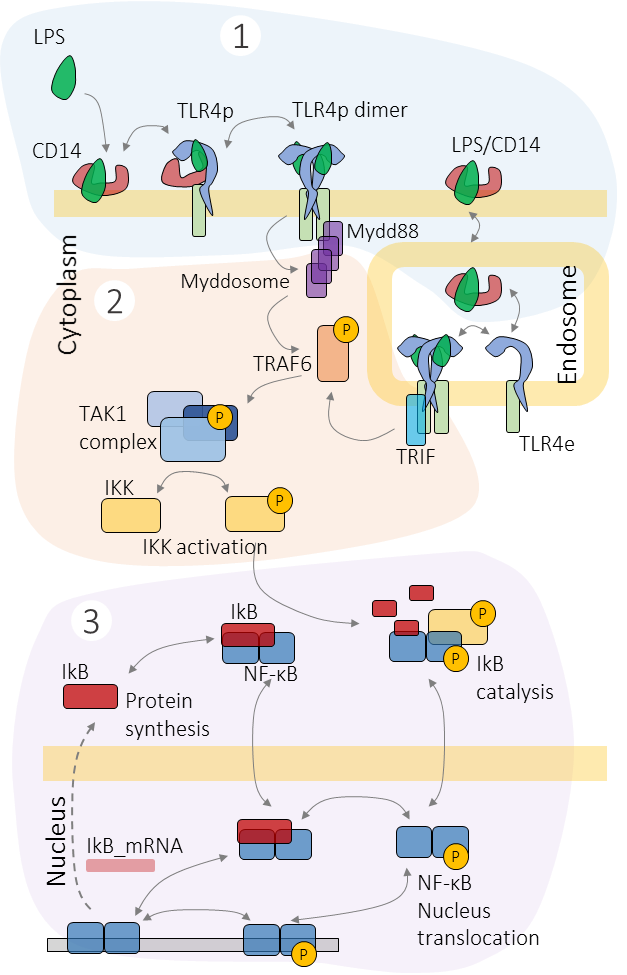
Fig S7. Module composition of TLR4 signaling used in the computational model.** 1) TLR4 module: LPS/CD14 binding and endosome translocation; 2) IKK module: MyD88 and TRIF activation that leads to IKK phosphorylation; 3) NF-κB module: degradation of IκB, an NF-κB inhibitor, and subsequent NF-κB translocation into the nucleus.

- 1. **TLR4 module (Module 1).**

This block of the model describes interactions of Toll receptor with its ligand – lipopolysaccharide, and endocytosis of TLR4/LPS complex by the cell (Fig S8). Mostly, reactions were accommodated from the work of ^7^. Additionally, we added a very important step of receptor dimerization (reaction describing the formation of the receptor complex from two TLR4/LPS complexes). It helped explain threshold in the receptor activation system (as described in the main text).

TLR4 signaling activation starts with binding of LPS by CD14 protein (r8) on the membrane of the macrophages. Then LPS/CD14/MD-2 complex become acceptable for TLR4 membrane receptor (r5, r6). (Here we followed by equations of Cheng et al. 2015, leaving outside the MD-2 protein). We tried not to change much in the kinetic parameters values for CD14 and TLR4 generation and degradation at the plasma membrane and within the cell (r1-3, r7-10, r13-15) because they were calculated from association constants measured on purified proteins. After binding of LPS, TLR4/LPS complex can be engulfed into endosome (r15), where it can be associated with the TRIF adaptor molecule or be degraded within the cell by other processes (r4).

**
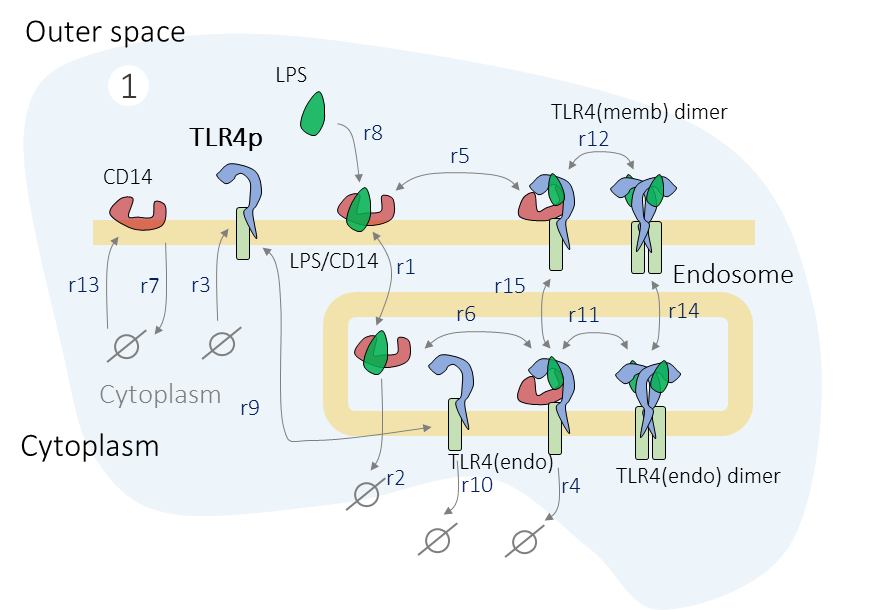
**

**Fig S8. TLR4 module (Module 1).** The addition of reactions describing Toll-like receptor dimer formation.

- - 1. **Receptor dimerization**

A significant gap in existing models is the absence of the receptor dimerization process description. This is a common and native property of the most receptors, that is intensively studied in the case of TLR4 receptor formation and activation ^8–10^. Dimer formation basically determines the ability of the receptor to self-induced transduction of activating signal ^11^. So, we added reactions (r11, r12) describing the dimer formation from two TLR4/LPS complexes and its dissociation (at the membrane or in the endosome). We used the doubled values for the kinetic parameters of dimer endocytosis compared to non-dimer complexes (r14). There was no available information about the kinetic parameters of the dimer formation, but we used the ratio of dimeric : monomeric forms of TLR4 receptor measured by Krüger and colleagues ^12^ to calculate the ratio of kinetic constants in reaction of dimerization $\frac{k_{1}}{k_{-1}}=\frac{2{Mono}^{2}}{Dimer}\approx40$ . We fitted kinetic rates for this equation keeping the observed ratio the same. New parameters influenced the TLR4/LPS dissociation dynamics, so we have had to change the dissociation constant rate for the TLR4/LPS complexes (r5, r6) (Table S1), previously fitted by ^7^

- 1. **IKK module (Module 2)**

Mathematically, this module (Fig S9) was significantly simplified as compared to known biological reactions within the molecular framework. The protein-protein interactions that transduce the signal from the activated receptor complex to the endpoint messenger molecules like NEMO/IKKα/β complex (here named simply IKK) represent the IKK module. The reaction for IKK activation module was taken primary from the works of Shark *et al*. 2015 and Cheng *et al*. 2015. In this view, we mathematically described the interaction of MyD88 protein adapter with plasma anchored TLR4/LPS dimer complex (r1-2) and the binding of TRIF adapter to endosomal TLR4/LPS dimer (r3).

**
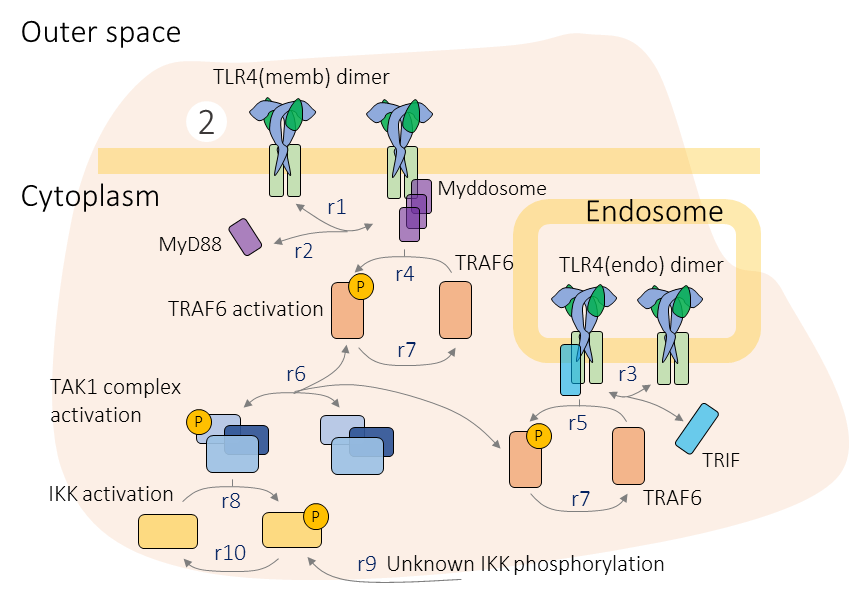
**

**Fig S9. IKK module (Module 2).** MyD88/TRIF activation pathways leading to the TAK1 mediated IKK activation. The process of constitutive “unknown” IKK phosphorylation creates a basal level of NF-κB translocation to the nucleus.

We accepted Cheng’s suggestion for describing myddosome formation at the TLR4 complex (six MyD88 molecules bind together at the same receptor site) in the form of Hill equation to the power of 3 (6 molecules form 3 complexes). Due to decrease of the receptor complex (TLR4/LPS) maximum concentration because of dimerization we had to decrease the K_D_ of this reaction to keep equation formally correct (r1). TRIF activation was kept with simple linear dynamics with modulation by TLR4(endo) dimer (r3). Activated MyD88 or TRIF complex modulate the phosphorylation of TRAF6 (r4, r5, r7), which in turn can induce the formation and activation of the TAK1/TAB1/2 complex (here named TAK1) (r6). The ubiquitination processes connected with TAK1 complex assembly was left outside the description, so we proposed a simple reaction describing the ability of activated TAK1 complex to phosphorylate IKK, thus activating reactions in the third ‘NF-κB module’ (r8, r10) (Yang et al 2013, Israël, 2010)

- - 1. **Basal constitutive NF-κB level in the nucleus**

Our experimental data suggested that Bone marrow derived macrophages had a high basal NF-κB level in the nucleus. If IKK is not activated at all, the total cellular pool of NF-κB is kept locked in the cytoplasm by IkB inhibitor. So, to describe a basal NF-κB translocation to the nucleus we have to add a reaction of low constitutive IKK phosphorylation. It was termed ‘unknown phosphorylation’ (r9). The existence of a low signal of phosphorylated IKK according to the western blot of non-activated macrophages supports this proposition. The exact source of basal NF-κB pre-activation was put outside the description. This could be connected with macrophage generation medium and the presence of GM-CSF ^14^, or cell culture conditions used in the experiments. Both of that causes are thought to be dependent on TRAF6 activation ^15^. We computationally fit the kinetic parameters for ‘unknown phosphorylation’ (r9) to achieve the exact basal NF-κB level at steady state condition of the system (Fig S10). Because of basal NF-κB level, initial values of other species were also recalculated accordingly.

^
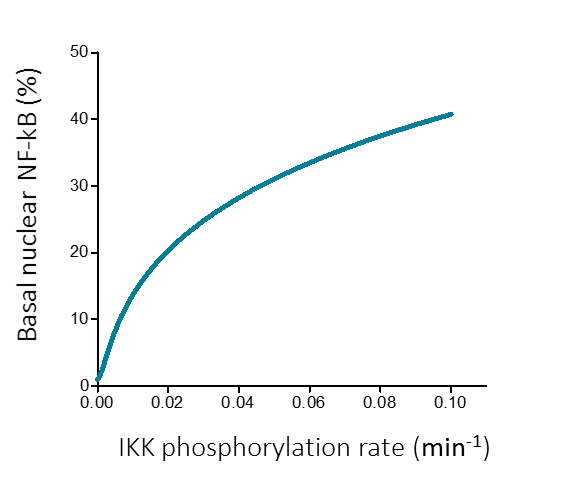
^

**Fig S10. Dependence of NF-κB basal level on ‘unknown IKK phosphorylation’ rate (r9**).

- 1. **NF-κB module (Module 3)**

First mathematical description of NF-κB module and its basic principles (Fig S11) were described for the culture of MEF cells by ^16^. It was slightly modified in further works in the next decade in works on different cell systems. Most of the equations for this module were taken from the work of Kearns *et al.* 2006 and Werner *et al*. 2008 and remained unchanged. The most important and new addition to these existing models were the reactions describing NF-κB phosphorylation by IKK complex and the NF-κB binding to the DNA.

The key feature of NF-κB module is a proteasome-dependent catalysis (r15-16) of IkB inhibitor that blocks NF-κB nuclear translocation by direct binding to NF-κB (r1). Activated IKK binds to the IκB and induces its degradation. When IκB degrades and NF-κB enters the nucleus it induces the synthesis of mRNA restoring concentration of IκB, that provides the negative feedback loop to the system (r24) ^17^. There are 3 isoforms of IκB protein – α, β and ε. IκBα is a major player in the negative feedback loop, other isoforms provide additional regulation. Here we used reactions describing the kinetics of all isoforms: the “–a” postfix in the reaction name means that this reaction is for IκBα, and there are two more of similar reactions (but with different kinetic parameters) for IκBβ and IκBε (Fig S11).


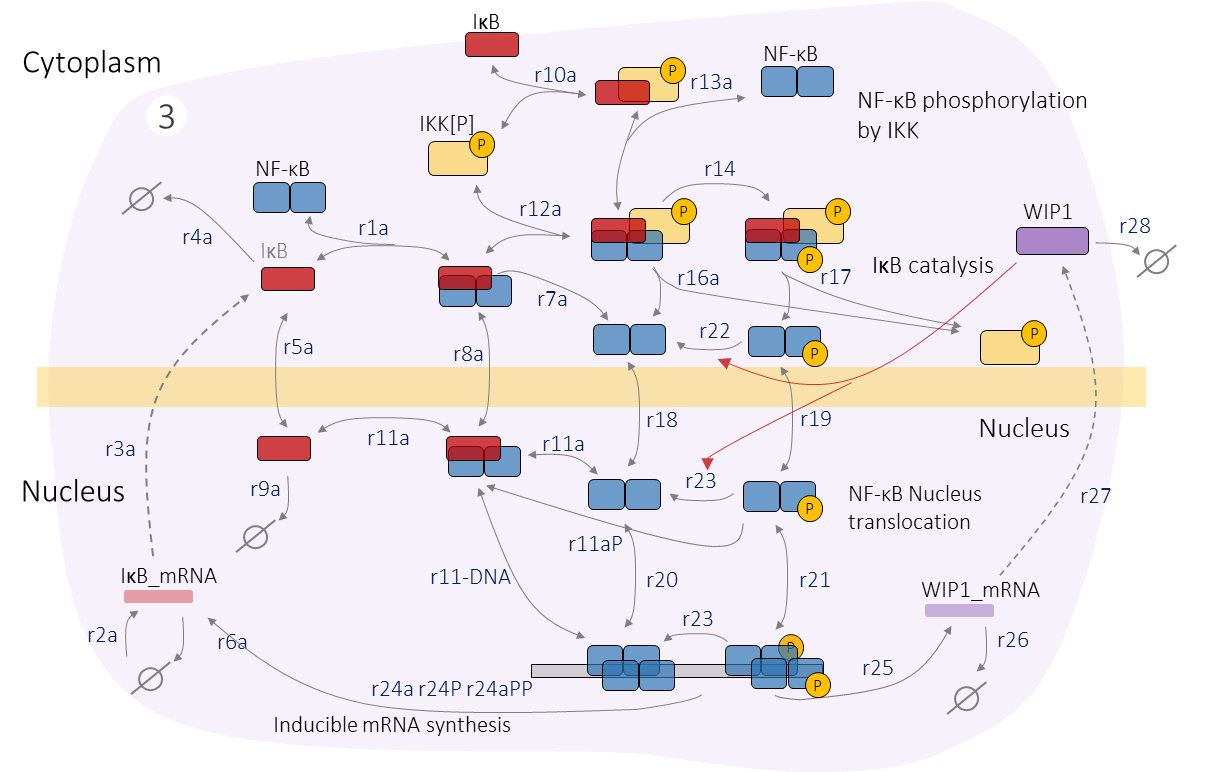


**Fig S11. NF-κB module (Module 3).** Phosphorylated IKK induces catalysis of IκB and translocation of NF-κB to the nucleus. Model additional describes IKK dependent NF-κB phosphorylation, dephosphorylation of NF-κB induced through WIP1 synthesis and binding of NF-κB to the DNA.

- - 1. **NF-κB phosphorylation**

A major new addition that we made to the existing models was an NF-κB phosphorylation process. Here we focused on the mathematical description of IKKα/β mediated phosphorylation of p65 subunit of NF-κB at ser 536 residue. Phosphorylation of transcription factors is thought to be the key regulatory mechanism of target genes induction ^18–20^. For simplicity, we chose an assumption that kinetic parameters in the reactions for both NF-κB or phosphorylated NF-κB[P] are the same. Thus, we fitted kinetic parameters in the reactions connected with NF-κB[P] only. The association of IκBα and NF-κB[P] in the cytoplasm, nucleus or DNA was taken to be zero, because cytosolic NF-κB[P] does not interact with IκBα (r1aP) ^19^.

We proposed that NF-κB phosphorylation happens upon IKK[P] binding to the IκBα/NF-κB complex(r14). After the IκBα catalysis, NF-κB[P] translocates to the nucleus. Here we also added reactions of spontaneous dephosphorylation in the nucleus and cytoplasm (r22, r23) We also found that simple constitutive dephosphorylation reactions weakly influenced NF-κB[P] dynamics and couldn’t explain experimental data of phosphorylated NF-κB kinetics. We proposed the existence of additional regulation via induced NF-κB dephosphorylation (as explained in the main text). The protein WIP1 was taken as an example of such process ^2^. It is induced by LPS stimulation ^21^ and therefore could explain observed kinetics of phosphorylated NF-κB. Upon inhibition of WIP1 synthesis, phosphorylated form of NF-κB reaches its high constant level ^2^, the same as predicted by our model calculations (S4 Fig). Induced dephosphorylation were added as a modulation to the r22 and r23 reactions. Also, we added reactions of WIP1 mRNA(r25-27) and protein (r28, r29) synthesis.

- - 1. **NF-κB to DNA binding**

To describe the process of NF-κB specific gene induction more correctly we added a step of NF-κB binding to DNA. That simple step is believed to be crucially important ^22^, (r20, r21, r11-DNA). Kinetic parameters were taken from the experimental work of ^23^. So, we included in our model a new species – part of NF-κB bound to DNA. The only proposition was made is that IkBvare not able to bind phosphorylated NF-κB at the DNA until it becomes dephosphorylated - the same as with cytoplasmic and nuclear forms of phosphorylated NF-κB. We also added reactions of induced IkB gene synthesis for phosphorylated form NF-κB, taking into account that reactions are nonlinear (r24).

**2. Computational simulations.**

All equations were solved numerically using Copasi package (//copasi.org) or MATLAB version R2008a (The MathWorks, Inc.) in the form of differential equations (Copasi file with model .cps**)** Experimental fitting was done using Nelder-Mead or Evolution programming algorithms (Copasi). Before calculation of time course kinetic of the model species, the model system was taken at steady state condition.

LPS concentration of 1000 ng/ml was recalculated as 0.1 uM of model value, proposing mean weight of LPS is 10kD [(Sigma, B5:055), Cheng *et al* 2015. Outer space volume was taken 1000 times larger than cellular volume (recalculation of the culture conditions that were used for LPS stimulation in the exact experiments gives 625-3000 values). For certain graphics we calculated kinetics of model species at different LPS concentrations (from 1e-08 to 10uM of model LPS).

- 1. **Mathematical fitting of kinetic parameters.**

All kinetic parameters used for computational calculations are presented in S4 Table. To accommodate kinetic parameters of the model for describing BMMϕ we used the experimental dynamics of NF-κB translocation as a starting point. We found that it is crucially important to use different concentrations of activating signal (LPS). A kinetics’ dependence on the LPS concentration creates certain restrictions for the fitted model kinetic parameters.

First, we fitted parameters for receptor dimerization in Module 1. (Table S1).

**Table S1. Amendments in kinetic parameters of Module 1 and addition of dimerization reactions.**

| **Reaction name (TLR4 module)** | **Description** | **Parameter fitted**  **(k_1_ Association / k_2_ Dissociation )** | | **Initial parameters** | |
| --- | --- | --- | --- | --- | --- |
| 5: Association of CD14LPS and TLR4 at the plasma membrane | CD14LPS + TLR4(memb) ↔ TLP4LPS(memb) | 5.543 | 0.295 | 5.543 | 0.027 |
| 6: Association of CD14LPS(endo) and TLR4 in the endosome | CD14LPS + TLR4(endo) ↔ TLP4LPS(endo) | 5.543 | 0.295 | 5.543 | 0.027 |
| 11: TLR4LPS(endo) dimerization | 2 * TLP4LPS(memb) ↔ dimerTLR4LPS(memb) | 21 | 0.5 | - | - |
| 12: TLR4LPS(memb) dimerization | 2 * TLP4LPS(memb) ↔ dimerTLR4LPS(memb) | 21 | 0.5 | - | - |
| 14: dimerTLR4LPS(memb) endocytosis | dimerTLR4LPS(memb) ↔ dimerTLR4LPS(endo) | 0.131 | 0.08 | Monomer  0.065 | Monomer  0.04 |

In module 2 we refitted all parameters the way to comply the following requirements. Firstly, NF-κB translocation and IκBα degradation kinetics (Fig S12, Fig S13). Secondly, to describe more biological values of the activated forms of intermediate kinases (Fig 3E, Table S2). We fitted parameters for NF-κB phosphorylation and its inhibition with induced WIP1 synthesis based on both dependences of NF-κB nucleus translocation and NF-κB phosphorylation kinetics (Fig S14, Table S3). Kinetic parameters are presented in Table S3. After changing the NF-κB/DNA dynamics we have had to change the rates for inducible IκBα mRNA Synthesis (r25), previously fitted by Sharp et al. ^24^

**Table S2. Fitting of the kinetic parameters of module 2 for BMMϕ.**

| **Reaction name (IKK module)** | **Description** | **Parameter fitted**  **Hill equation**  **V, h (power) , K_D_** | | | | **Initial parameters** | | |
| --- | --- | --- | --- | --- | --- | --- | --- | --- |
| 1: MYD88 Activation by dimerTLR4LPS(memb) | MYD88 → MYD88* ; dimerTLR4LPS (memb) | 52.90 | 3 | | 1.75E-03 | 150 | 3 | 0.012 |
|  |  | **(k_1_ Association / k_2_ Dissociation )** | | | | **Initial parameters (k_1_ / k_2_ )** | | |
| 2: MYD88 Deactivation | MYD88* → MYD88 | 150.7 | | - | | 2200 | - | |
| 3: TRIF activation by dimerTLR4LPS(endo) | TRIF ↔ TRIF* ; dimerTLR4LPS (endo) | 250 | | 1.45 | | 18 | 0.04 | |
| 4: TRAF6 activation by MYD88 | TRAF6 → TRAF6[P] ; MyD88* | 200 | | - | | 550 | - | |
| 5: TRAF6 activation by TRIF | TRAF6 → TRAF6[P] ; TRIF* | 50 | | - | | 16 | - | |
| 6: TAK1 activation by TRAF6 | TAK1 ↔ TAK1[P] ; TRAF[P] | 8 | | 3.87 | | 0.098 | 2.5 | |
| 7: TRAF6 deactivation | TRAF6[P] → TRAF6 | 10 | | - | | 18 | - | |
| 8: IKK activation by TAK1 | IKK → IKK[P] ; TAK1[P] | 200 | | - | | 1000 | - | |
| 9: IKK activation by unknown signal | IKK → IKK[P] | 0.038 | | - | | - | - | |
| 10: IKK deactivation | IKK[P] → IKK | 6 | - | | | 0.9 | - | |


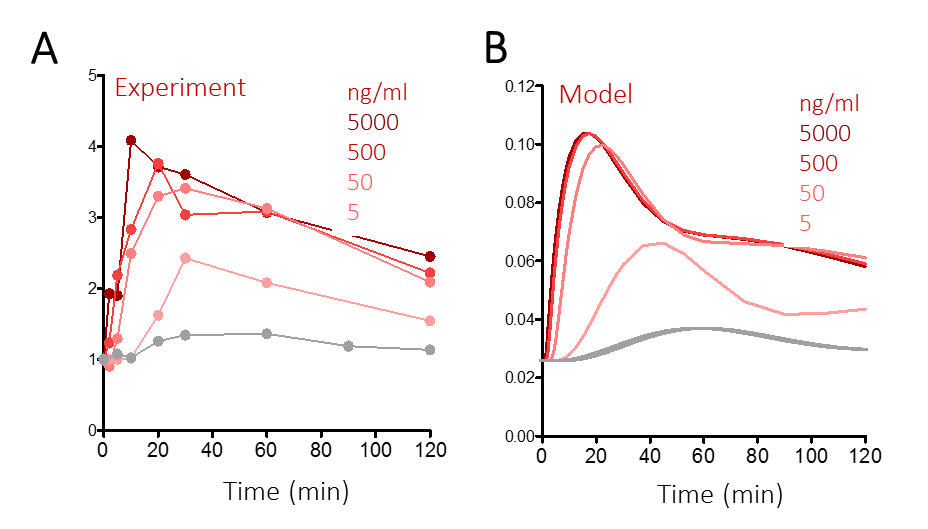


**Fig S12. NF-κB nuclear translocation kinetics**. **A –** Experimental measurements of the NF-κB signal in the nucleus of BMMϕ at different concentrations of LPS (from 5 ng/ml to 5000 ng/ml). Data normalized to the NF-κB signal in the nucleus of non-activated cells. **B –** In silico calculations of the NF-κB nuclear translocation at the same LPS concentrations.


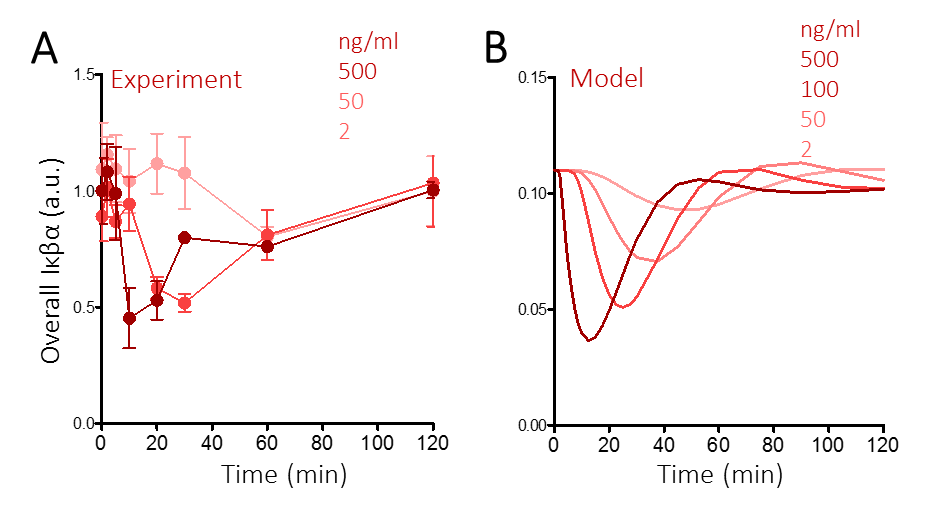


**Fig S13. IκBα degradation kinetics**. **A –** Experimental measurements of the cellular IκBα signal in the BMMϕ at different concentrations of LPS (from 2 ng/ml to 500 ng/ml). Data normalized to the cellular IκBα signal in non-activated cells. **B –** In silico calculations of the cellular IκBα concentration kinetics at the same LPS concentrations.

**Table S3. The addition of new reactions of NF-κB phosphorylation into module 3.**

| **Reaction name (NF-κB module)** | **Description** | **Parameter fitted**  **(k_1_ Association / k_2_ Dissociation )** | | **Initial parameters**  **(k_1_ / k_2_ )** | |
| --- | --- | --- | --- | --- | --- |
| 22: NFkB[P] dephosphorylation | NFkB[P] → NFkB | 1.00E-03 | - | - |  |
| 23: NFkB[P](nuc) dephosphorylation | NFkB[P](nuc) → NFkB(nuc) | 0.031 | - | - |  |
| 23 - DNA: NFkBDNA[P](nuc) dephosphorylation | NFkBDNA[P](nuc) → NFkBDNA(nuc) | 0.079 |  |  |  |
| 24a: Inducible IκBα mRNA Synthesis (NFkBDNA(nuc)) | 2 * NFkBDNA(nuc) → IkBa_mRNA + 2 * NFkBDNA(nuc) | 0.3778 | - | - |  |
| 24aP: Inducible IkBa mRNA Synthesis (NFkBDNA[P](nuc)) combinations | NFkBDNA[P](nuc) + NFkBDNA(nuc) → IkBa_mRNA + NFkBDNA[P](nuc) + NFkBDNA(nuc) | 0.3778 | - | - |  |
| 24aPP: Inducible IkBa mRNA Synthesis (NFkBDNA[P](nuc)) | | | | | |
| **(**WIP1 **module)** |  |  |  |  |  |
| 25: Inducible WIP1 mRNA Synthesis DNA | Ø →WIP1_mRNA; 2 * NFkBDNA | 0.681 | - | - |  |
| 26: Inducible WIP1 mRNA Synthesis DNA combinations | NFkBDNA[P](nuc) + NFkBDNA(nuc) -> WIP1_mRNA + NFkBDNA[P](nuc) + NFkBDNA(nuc) | 0.681 | - | - |  |
| 27: WIP1 mRNA degradation | WIP1_mRNA → Ø | 0.093 | - | - |  |
| 29: WIP1 degradation | WIP1→ Ø | 0.1 |  |  |  |
| 28: WIP1 synthesis | WIP1_mRNA → WIP1 + WIP1_mRNA | 0.2 | - | - |  |


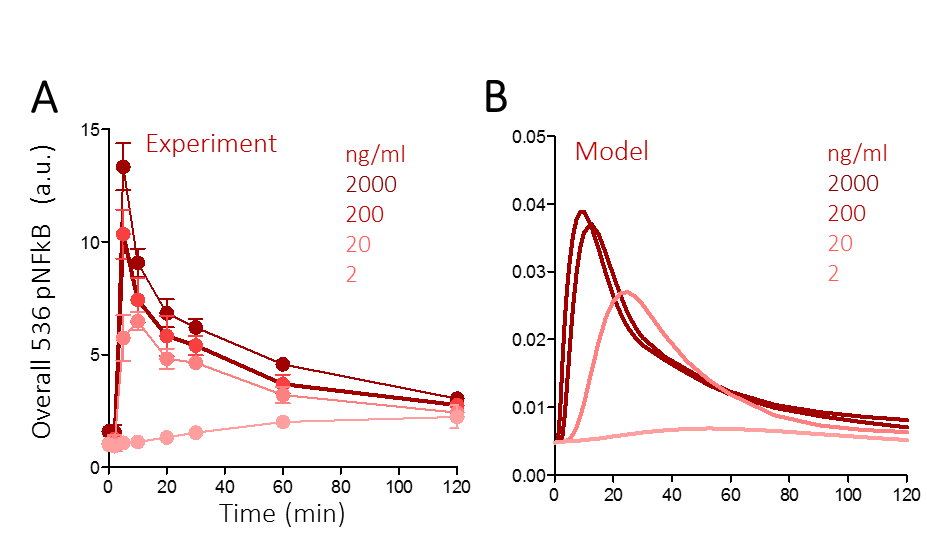


**Fig S14. NF-κB phosphorylation kinetics**. **A –** Experimental measurements of the NF-κB phosphorylation (p65 subunit at ser 536) of BMMϕ at different concentrations of LPS (from 2 ng/ml to 2000 ng/ml). Data normalized to the NF-κB phosphorylation (p65 subunit at ser 536) in non-activated cells. **B –** In silico calculations of the NF-κB phosphorylation at the same LPS concentrations (Model concentration of 1uM were taken equal to 1000ng/ml).

- 1. **Importance of NF-κB-to-DNA interaction in the suggested model**

Previous models used an assumption that when NF-κB enters the nucleus it initiates transcription by its own, leaving aside a complicated processes of NF-κB binding to DNA and polymerase complex formation ^24,25^. Other authors implemented an artificial delay (time delay as an additional parameter) in mRNA transcription relative to the NF-κB nuclear translocation kinetics ^7,26,27^. Although the NFkB-DNA binding kinetics is very fast ^23,28,29^, in-depth analysis showed that to describe the process of NF-κB specific gene induction more correctly, it is important to add a direct step of NF-κB binding to DNA. Such process can provide the intended delay in the induction of gene transcription. Direct NF-κB binding to DNA let us better describe synthesis mRNA of NF-κB target genes like TNFα (Fig S15)

**
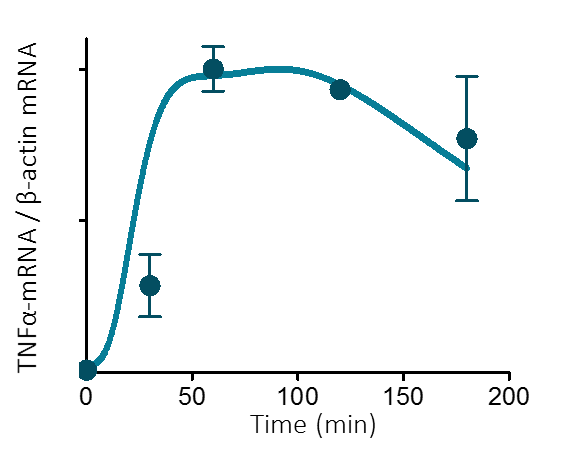
**

**Fig S15. TNFα gene mRNA transcription kinetics.** Kinetics of TNFα mRNA synthesis (dots) measured with RTPCR and model prediction (line) in case if only DNA-bound NF-κB form induces TNFα gene synthesis.


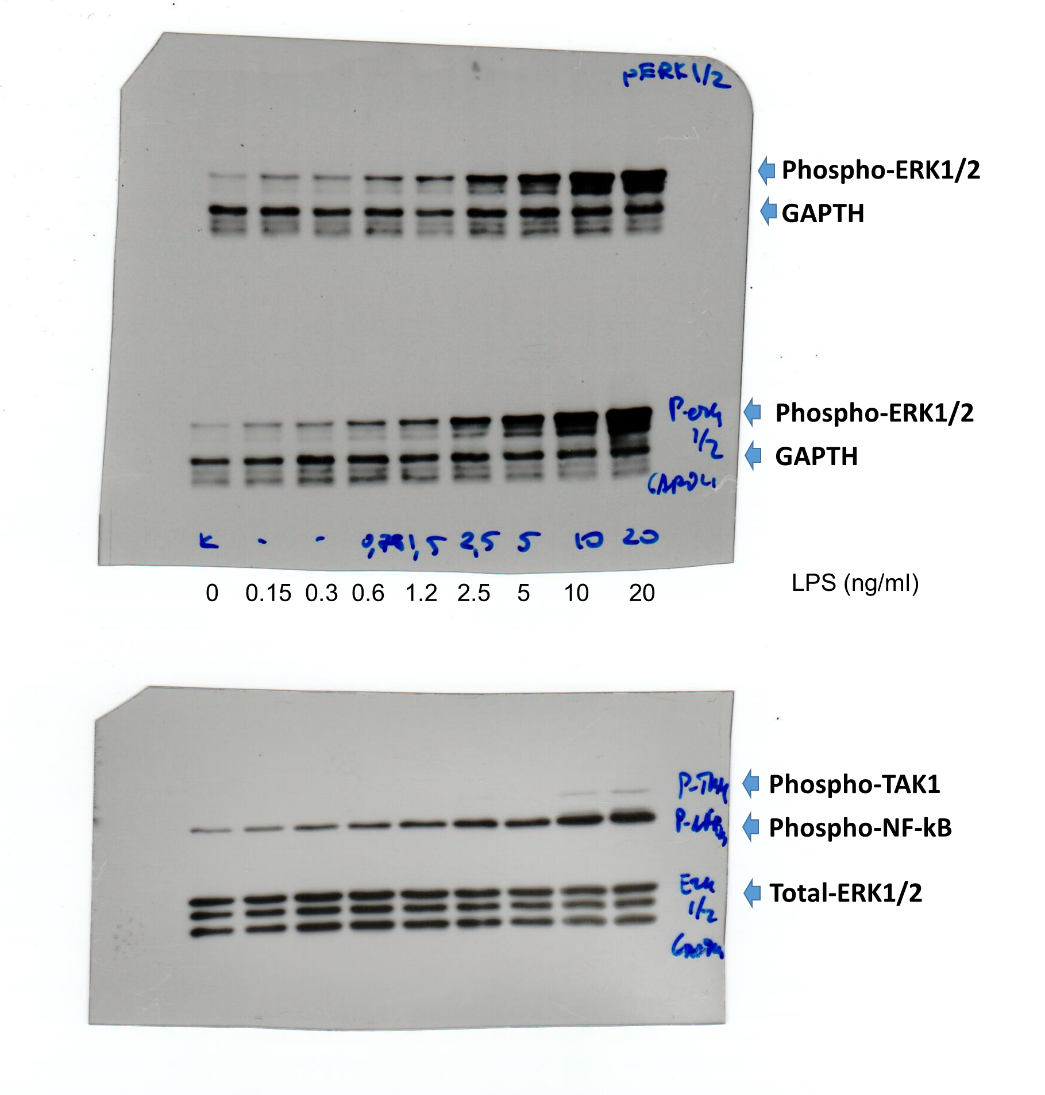


**Fig S16. Blots used in the study**

**
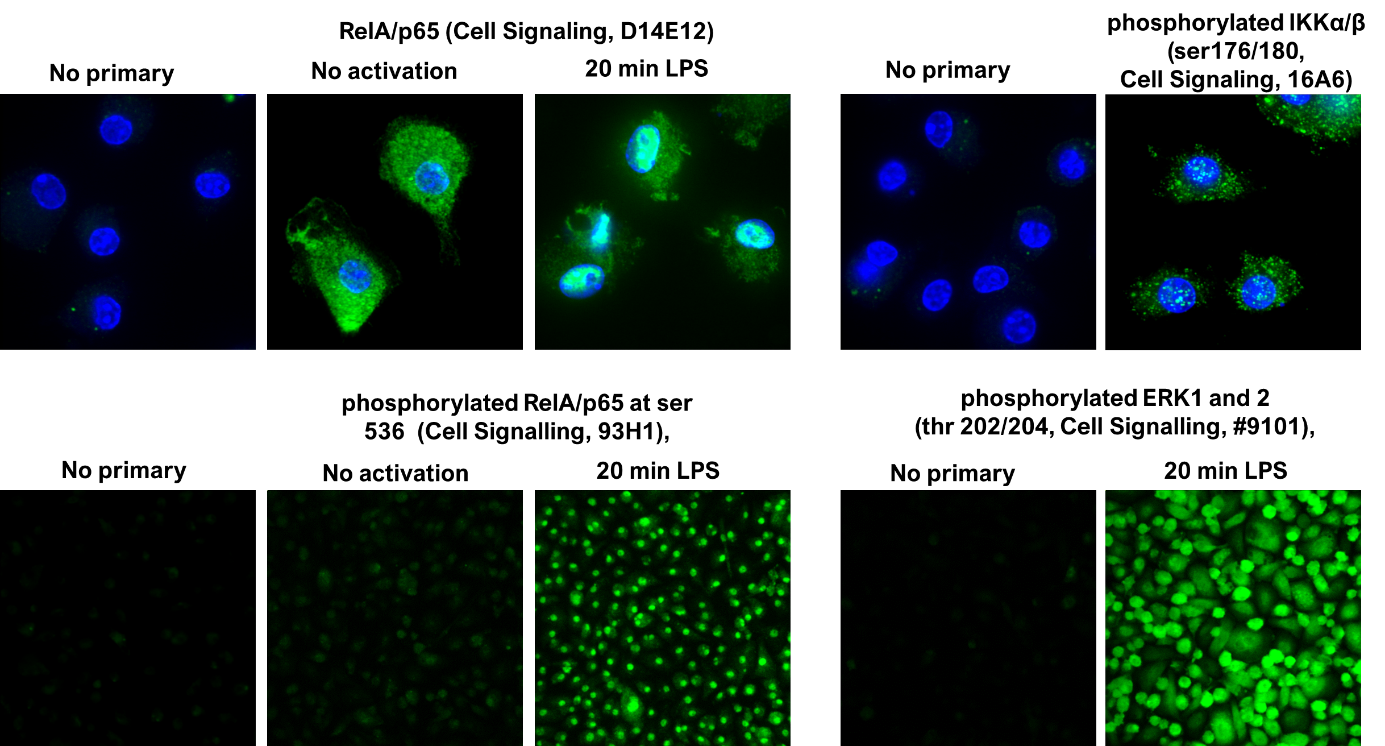
**

**Fig S17.** Representative staining and a no primary control for antibodies used in the study.

**Supplement references**

1. Helft, J. *et al.* GM-CSF Mouse Bone Marrow Cultures Comprise a Heterogeneous Population of CD11c+MHCII+ Macrophages and Dendritic Cells. *Immunity* **42,** 1197–1211 (2015).

2. Chew, J. *et al.* WIP1 phosphatase is a negative regulator of NF-kappaB signalling. *Nat. Cell Biol.* **11,** 659–66 (2009).

3. Oda, K. & Kitano, H. A comprehensive map of the toll-like receptor signaling network. *Mol. Syst. Biol.* **2,** 2006.0015 (2006).

4. Kawai, T. & Akira, S. The role of pattern-recognition receptors in innate immunity: update on Toll-like receptors. *Nat. Immunol.* **11,** 373–84 (2010).

5. Kawai, T. & Akira, S. TLR signaling. *Semin Immunol* **19,** 24–32 (2007).

6. Takeda, K. & Akira, S. Toll-like receptors in innate immunity. *Int. Immunol.* **17,** 1–14 (2005).

7. Cheng, Z., Taylor, B., Ourthiague, D. R. & Hoffmann, A. Distinct single-cell signaling characteristics are conferred by the MyD88 and TRIF pathways during TLR4 activation. *Sci. Signal.* **8,** 1–13 (2015).

8. Park, B. S. *et al.* The structural basis of lipopolysaccharide recognition by the TLR4-MD-2 complex. *Nature* **458,** 1191–5 (2009).

9. Bovijn, C. *et al.* Identification of interaction sites for dimerization and adapter recruitment in toll/interleukin-1 receptor (TIR) domain of toll-like receptor 4. *J. Biol. Chem.* **287,** 4088–4098 (2012).

10. Tsukamoto, H., Fukudome, K., Takao, S., Tsuneyoshi, N. & Kimoto, M. Lipopolysaccharide-binding protein-mediated Toll-like receptor 4 dimerization enables rapid signal transduction against lipopolysaccharide stimulation on membrane-associated CD14-expressing cells. *Int. Immunol.* **22,** 271–280 (2010).

11. Park, B. S. & Lee, J.-O. Recognition of lipopolysaccharide pattern by TLR4 complexes. *Exp. Mol. Med.* **45,** e66 (2013).

12. Krüger, C. L., Zeuner, M., Cottrell, G. S., Widera, D. & Heilemann, M. Quantitative single-molecule imaging of TLR4 reveals ligand-specific receptor dimerization. *Sci. Signal.* **10,** eaan1308 (2017).

13. Israël, A. The IKK Complex, a Central Regulator of NF-κB Activation. *Cold Spring Harb. Perspect. Biol.* **2,** 14 (2010).

14. Ebner, K., Bandion, A., Binder, B. R., Martin, R. De & Schmid, J. A. GMCSF activates NF- ␬ B via direct interaction of the GMCSF receptor with I ␬ B kinase ␤. **102,** 192–199 (2003).

15. Meads, M. B., Li, Z.-W. & Dalton, W. S. A novel TNF receptor-associated factor 6 binding domain mediates NF-kappa B signaling by the common cytokine receptor beta subunit. *J. Immunol.* **185,** 1606–1615 (2010).

16. Hoffmann, A., Levchenko, A., Scott, M. L. & Baltimore, D. The IkB - NFkB Signaling Module : Temporal Control and Selective Gene Activation. *Science (80-. ).* **1241,** (2002).

17. Fagerlund, R. *et al.* Anatomy of a negative feedback loop: The case of IκB. *J. R. Soc. Interface* **12,** (2015).

18. Hochrainer, K., Racchumi, G. & Anrather, J. Site-specific phosphorylation of the p65 protein subunit mediates selective gene expression by differential NF-kB and RNA polymerase II promoter recruitment. *J. Biol. Chem.* **288,** 285–293 (2013).

19. Sasaki, C. Y., Barberi, T. J., Ghosh, P. & Longo, D. L. Phosphorylation of Re1A/p65 on serine 536 defines an IkBa- independent NF-kB pathway. *J. Biol. Chem.* **280,** 34538–34547 (2005).

20. Viatour, P., Merville, M. P., Bours, V. & Chariot, A. Phosphorylation of NF-κB and IκB proteins: Implications in cancer and inflammation. *Trends Biochem. Sci.* **30,** 43–52 (2005).

21. Tan, X. *et al.* Wip1 phosphatase involved in lipopolysaccharide-induced neuroinflammation. *J. Mol. Neurosci.* **51,** 959–966 (2013).

22. Wan, F. & Lenardo, M. J. Specification of DNA Binding Activity of NF- B Proteins. *Cold Spring Harb. Perspect. Biol.* **1,** a000067–a000067 (2009).

23. Bergqvist, S. *et al.* Kinetic enhancement of NF-kappaBxDNA dissociation by IkappaBalpha. *Proc. Natl. Acad. Sci. U. S. A.* **106,** 19328–19333 (2009).

24. Sharp, G. C., Ma, H., Saunders, P. T. K. & Norman, J. E. A Computational Model of Lipopolysaccharide-Induced Nuclear Factor Kappa B Activation: A Key Signalling Pathway in Infection-Induced Preterm Labour. *PLoS One* **8,** (2013).

25. Selvarajoo, K. Discovering differential activation machinery of the Toll-like receptor 4 signaling pathways in MyD88 knockouts. *FEBS Lett.* **580,** 1457–1464 (2006).

26. Werner, S. L. Stimulus Specificity of Gene Expression Programs Determined by Temporal Control of IKK Activity. *Science (80-. ).* **309,** 1857–1861 (2005).

27. Covert, M. W., Leung, T. H., Gaston, J. E. & Baltimore, D. Achieving stability of lipopolysaccharide-induced NF-kappaB activation. *Science* **309,** 1854–1857 (2005).

28. Phelps, C. B., Sengchanthalangsy, L. L., Malek, S. & Ghosh, G. Mechanism of κB DNA binding by Rel/NF-κb dimers. *J. Biol. Chem.* **275,** 24392–24399 (2000).

29. Bosisio, D. *et al.* A hyper-dynamic equilibrium between promoter-bound and nucleoplasmic dimers controls NF-kappaB-dependent gene activity. *EMBO J.* **25,** 798–810 (2006).
